# Phase-Coherent Charge Transport through a Porphyrin Nanoribbon

**DOI:** 10.1021/jacs.3c02451

**Published:** 2023-07-07

**Authors:** Zhixin Chen, Jie-Ren Deng, Songjun Hou, Xinya Bian, Jacob L. Swett, Qingqing Wu, Jonathan Baugh, Lapo Bogani, G. Andrew D. Briggs, Jan A. Mol, Colin J. Lambert, Harry L. Anderson, James O. Thomas

**Affiliations:** † Department of Materials, University of Oxford, Parks Road, Oxford OX1 3PH, U.K.; ‡ Department of Chemistry, Chemistry Research Laboratory, University of Oxford, Oxford OX1 3TA, U.K.; § Department of Physics, Lancaster University, Lancaster LA1 4YB, U.K.; ∥ Institute for Quantum Computing, University of Waterloo, Waterloo, Ontario N2L 3G1, Canada; ⊥ School of Physical and Chemical Sciences, Queen Mary University, London E1 4NS, U.K.

## Abstract

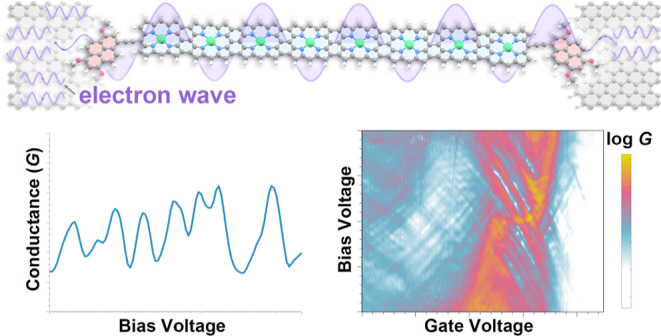

Since the early days of quantum mechanics, it has been known that electrons behave simultaneously as particles and waves, and now quantum electronic devices can harness this duality. When devices are shrunk to the molecular scale, it is unclear under what conditions does electron transmission remain phase-coherent, as molecules are usually treated as either scattering or redox centers, without considering the wave–particle duality of the charge carrier. Here, we demonstrate that electron transmission remains phase-coherent in molecular porphyrin nanoribbons connected to graphene electrodes. The devices act as graphene Fabry–Pérot interferometers and allow for direct probing of the transport mechanisms throughout several regimes. Through electrostatic gating, we observe electronic interference fringes in transmission that are strongly correlated to molecular conductance across multiple oxidation states. These results demonstrate a platform for the use of interferometric effects in single-molecule junctions, opening up new avenues for studying quantum coherence in molecular electronic and spintronic devices.

## Introduction

The ability to harness and exploit coherence at the nanoscale is crucial for emerging quantum technologies being developed in research areas across engineering, chemistry, and condensed-matter physics, and it may also play a role in electron transfer in biomolecular systems.^1,2^ Single-molecule devices are an excellent platform to study quantum-coherent phenomena because molecular structures are atomically defined,^3^ and recently bottom-up synthesized graphene^4^ and molecular nanoribbons^5^ have attracted attention in quantum information processing due to their low dimensionality and associated topological states. Most studies of single molecules in junctions have focused on observing quantum phenomena, such as quantum interference (QI), in two-terminal devices by comparing transport properties of homologous series of molecules^6,7^ and averaging data such that details of different transport mechanisms are lost, rather than manipulating and studying QI within the same molecule.^8,9^ The ability to measure and tune the transport properties of the same single-molecule device, by changing the gate potential, magnetic field, and temperature, is necessary to understand how the different transport mechanisms that arise from molecular, electrode, and molecule–electrode hybrid states all come together to contribute to the device conductance. Studies of this nature can answer questions such as: How can electron transmission be shown to be phase coherent? What molecule–electrode coupling regime^10^ is required for this to be the case? The understanding gained from these studies feeds into one of the ultimate goals of single-molecule electronics, which is to integrate molecules and nanoribbons,^11^ one-by-one, into solid-state devices with some functionality that exploits the quantum properties of an individual molecule.

Graphene devices are an ideal platform for investigating phase-coherent phenomena in charge transport.^12−14^ The spatial confinement and long coherence lengths of electrons in graphene mean that devices display a range of quantum-coherent features, such as electronic Fabry–Pérot (FP) interferometry.^15,16^ Furthermore, there are established routes to interface molecules, including biomolecules, with graphene through π-stacking interactions^17^ to generate electrostatically gated single-molecule graphene junctions, enabling temperature and magnetic-field-dependent measurements with a high operating frequency.^10,18^ This is facilitated by the weaker screening by graphene of the gate electric field, compared with bulk 3D metallic electrodes used in traditional molecular junctions,^19,20^ and by the electronic band structure of graphene, which makes it possible to differentiate between the contributions to transport from graphene and molecular states.^21^

**Figure 1:**
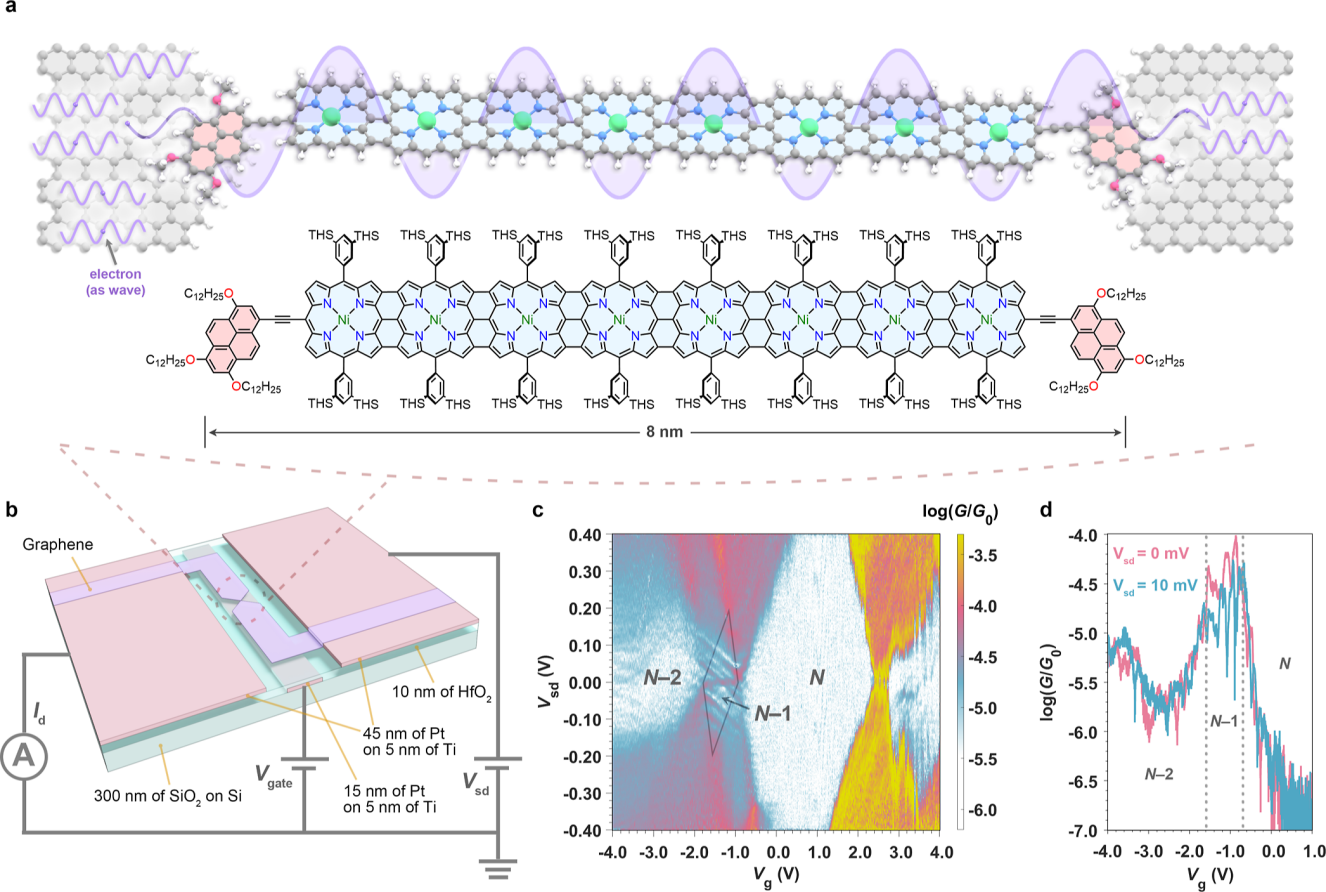
Phase-coherent electron transport through a porphyrin nanoribbon-graphene device. (a) Simplified scheme of phase-coherent electron transport through a graphene–**Ni-FP8** porphyrin nanoribbon–graphene junction (top) and full chemical structure of **Ni-FP8** (bottom, THS = trihexylsilyl). In the top panel, solubilizing groups on porphyrins (3,5-*bis*(trihexylsilyl)phenyl) and pyrenes (dodecyloxy) have been omitted for clarity. (b) Device architecture. The pink rectangular area in the middle represents the local platinum gate electrode under a 10 nm layer of HfO_2_ (transparent light blue); the rectangular areas (pink) at both ends represent the source and drain platinum electrodes, which are in contact with the bowtie-shaped graphene (purple). (c) Differential conductance (*G* = d*I*_sd_/d*V*_sd_) map measured as a function of bias voltage (*V*_sd_) and gate voltage (*V*_g_) and (d) differential conductance as a function of *V*_g_ at *V*_sd_ = 0 mV (pink curve) and *V*_sd_ = 10 mV (blue curve) for **Ni-FP8** device 1 at 4.2 K. The conductance is plotted in a logarithmic scale as the ratio to conductance quantum *G*_0_ = 2*e*^2^/*h*, where *e* is the elementary charge and *h* is Planck’s constant. The *N* – 1 charge state is highlighted by a gray diamond.

In this work, we study charge transport through porphyrin nanoribbon–graphene devices at cryogenic temperatures (Figure 1a) and demonstrate that conductance measurements as functions of (source–drain) bias voltage (*V*_sd_), gate voltage (*V*_g_), and temperature exhibit a wide range of QI phenomena such as FP and Kondo resonances. Our results demonstrate that an 8 nm porphyrin octamer nanoribbon sustains phase-coherent electron transmission that can be tuned electrostatically and is highly oxidation-state dependent. Overall, we reveal a comprehensive picture of different quantum-coherent phenomena that arise in molecule–graphene junctions.

## Results

We focus on charge transport through an edge-fused nickel(II) porphyrin octamer (**Ni-FP8**) device (Figure 1a). **Ni-FP8** has a length of 8 nm, and we term it a porphyrin nanoribbon, because it has a similar aspect ratio and electronic structure to a graphene nanoribbon.^22,23^ The synthesis of the **Ni-FP8** nanoribbon is described in the Experimental Section and Supporting Information Section S2. Most previous transport studies with porphyrin oligomers have used zinc(II) complexes,^24,25^ but in this case nickel was used to facilitate the synthesis and to reduce the energy of the HOMO, thus enhancing the chemical stability of the ribbon. The porphyrin octamer **Ni-FP8** is diamagnetic in its neutral oxidation state, because the nickel(II) ions have a low-spin d^8^ electron configuration. The molecules are soluble due to the presence of *bis*(trihexylsilyl)phenyl groups that decorate the porphyrin units; these groups prevent aggregation and improve the likelihood of obtaining transport data dominated by a single nanoribbon.^26^ The fused porphyrin octamer core is functionalized with tridodecyloxypyrene (TDP) anchor groups at both ends, to bind to the graphene source and drain electrodes via π-stacking. An idealized view of the junction is displayed in Figure 1a. In reality, the porphyrin core may overlap partially with the graphene; however, we expect the molecule–electrode electronic coupling to be primarily mediated by π–π stacking between pyrene groups and graphene, due to the presence of bulky trihexylsilyl groups on the porphyrins that hinder direct interaction of the porphyrins with the graphene.

A schematic of the device is shown in Figure 1b. The fabrication procedure is detailed in the Experimental Section and outlined briefly here. First, a gate electrode (Ti/Pt) is deposited onto a Si/SiO_2_ substrate by electron-beam evaporation and then covered with an ALD-grown dielectric layer of 10 nm of HfO_2_. The thin, high-κ HfO_2_ layer provides a large electrostatic coupling between the chemical potentials of the molecular states and the gate potential, typically larger than 0.1 eV/V.^27^ The large coupling, combined with a dielectric breakdown voltage for the HfO_2_ of *V*_g_ ≈ ± 5 V, allows multiple molecular charge states to be measured. Platinum source and drain electrodes are deposited next, and then CVD-grown graphene is transferred onto the whole substrate. The graphene is patterned into a bow-tie-shaped constriction (shown in Figure 1b) with a width of 100 nm at the narrowest point using a combination of electron-beam lithography and O_2_-plasma etching. We have improved a previous fabrication procedure^28^ by using a Z-shaped pattern for the graphene, to reduce tension in the constriction, and using a positive, rather than negative, photoresist to reduce contamination. A combination of these two changes may contribute to the stronger electronic coupling we observe in this work compared to previous studies.^17^

Feedback-controlled electroburning is used to convert the bowtie-shaped constrictions into nanometer-spaced graphene electrodes (see the Supporting Information for electroburning curves).^29,30^ The source-drain electrode spacing (i.e., the width of the nanogap) can be determined by fitting the *I*_sd_–*V*_sd_ trace, measured after electroburning, to the Simmons model.^31^ This typically yields values of 1.0–2.5 nm;^17^ however, for the **Ni-FP8** devices described below, the tunneling current was too low for Simmons fitting, leading us to estimate a lower bound for the gap size of >2.5 nm for these devices.

The porphyrin nanoribbons are interfaced with the electroburnt nanogaps by drop-casting a solution of **Ni-FP8** in toluene (1 μM) directly onto the devices. The tunneling current through the devices before and after the drop-casting are compared, and only when there are new regions of high tunneling current after deposition that are at least an order of magnitude higher than the baseline are the devices wire-bonded and cooled for detailed measurements (see Supporting Information Figure S3-1 for this comparison). We discuss transport through two **Ni-FP8** devices in the main text (device 1 and device 2) and also present data from two shorter porphyrin oligomers shown in Supporting Information Figure S3-6: one with a zinc porphyrin monomer (**Zn-P1**) one with an edge-fused porphyrin trimer (**Zn-FP3**) that display the same evidence for phase-coherent charge transport outlined below.

### Charge Transport Measurements

The full differential conductance map (*G*_sd_ = d*I*_sd_/d*V*_sd_) of the porphyrin nanoribbon **Ni-FP8** device 1, measured at 4.2 K, displays several Coulomb diamonds and associated resonant tunneling regions (Figure 1c). We calculate the coupling of the molecular levels to the gate and source potentials from the slopes of the Coulomb diamonds, as α_g,mol_ = 0.22 eV/V and α_s,mol_ = 0.65 eV/V respectively, giving the fraction of each applied potential that the molecular levels shift by.^20^ As the slopes of the resonant tunneling regions are the same for all transitions, and the Coulomb diamonds close at zero bias, the tunneling current is dominated by transport through a single molecular nanoribbon uncoupled to any nearby molecules.^32,33^ We assign the diamond at *V*_g_ = 0 as the *N* state (*N* being the number of electrons on the molecule when it is neutral), as this diamond has a large addition energy, at *E*_add_ = 0.7 eV. There is a zero-bias conductance peak in the neighboring diamond between *V*_g_ ≈−0.7 V and *V*_g_ ≈ −1.4 V. This peak is consistent with the experimental signature of a Kondo resonance (discussed in detail below) that results from screening of an unpaired spin on the nanoribbon by electrons within the graphene electrodes.^34,35^ The observation of a Kondo resonance is consistent with assigning this smaller diamond to the odd *N* – 1 state (i.e., the molecule is the radical cation, **Ni-FP8**^**+**^, in this Coulomb diamond), confirming that the larger diamond at *V*_g_ = 0 is the even *N* state, as the number of electrons on the molecule differs by one between adjacent Coulomb diamonds.^27^ The two sequential transport regions with broad edges that flank the *N* – 1 diamond are then the *N* – 1/*N* charge transition (*V*_g_ ∼ −0.7 V) and the *N* – 2/*N* – 1 transition (*V*_g_ ∼ −1.4 V). Finally, the highly conductive region at positive *V*_g_ is the *N*/*N* + 1 charge transition.

Considering the low measurement temperature (4.2 K, *k*_B_*T* = 0.4 meV), the poor definition in the boundaries of the Coulomb diamonds (fwhm of Coulomb peaks ∼14 meV, see Supporting Information Figure S4-2) is attributed to lifetime broadening that results from intermediate molecule–electrode coupling, a regime consistent with the appearance of the Kondo resonance.^36,37^ The broad diamond edges suggest that there are large regions where the molecular charge state is not well defined, in contrast with a device in the weakly coupled regime (commonly observed for molecules π-stacked to graphene electrodes^17,20,38^), where conductance occurs only within the sequential transport regions (when the chemical potentials of molecular transitions lie within the bias window, neglecting coherent resonant tunneling) separated by Coulomb diamonds. In Figure 1c,d, we observe off-resonance transport features showing that, even at *V*_sd_ = 10 mV (away from the Kondo peak), the conductance is not completely suppressed. Instead, the conductance remains above the noise except within the *N* diamond, indicating that there are significant contributions from off-resonant phase-coherent transport around the *N* – 1/*N* and *N* – 2/*N* – 1 transitions of the intermediately coupled nanoribbon device.^36^ As the hybridization between molecule and electrode increases with electronic coupling, transport through an intermediately coupled molecular junction can only be fully understood by considering the entire graphene–nanoribbon–graphene system. We discuss the mixture of effects that arise from this holistic approach by initially focusing on phase-coherent transport within the graphene channel and then on molecular transport.

### Graphene-Dominated Interference

**Figure 2:**
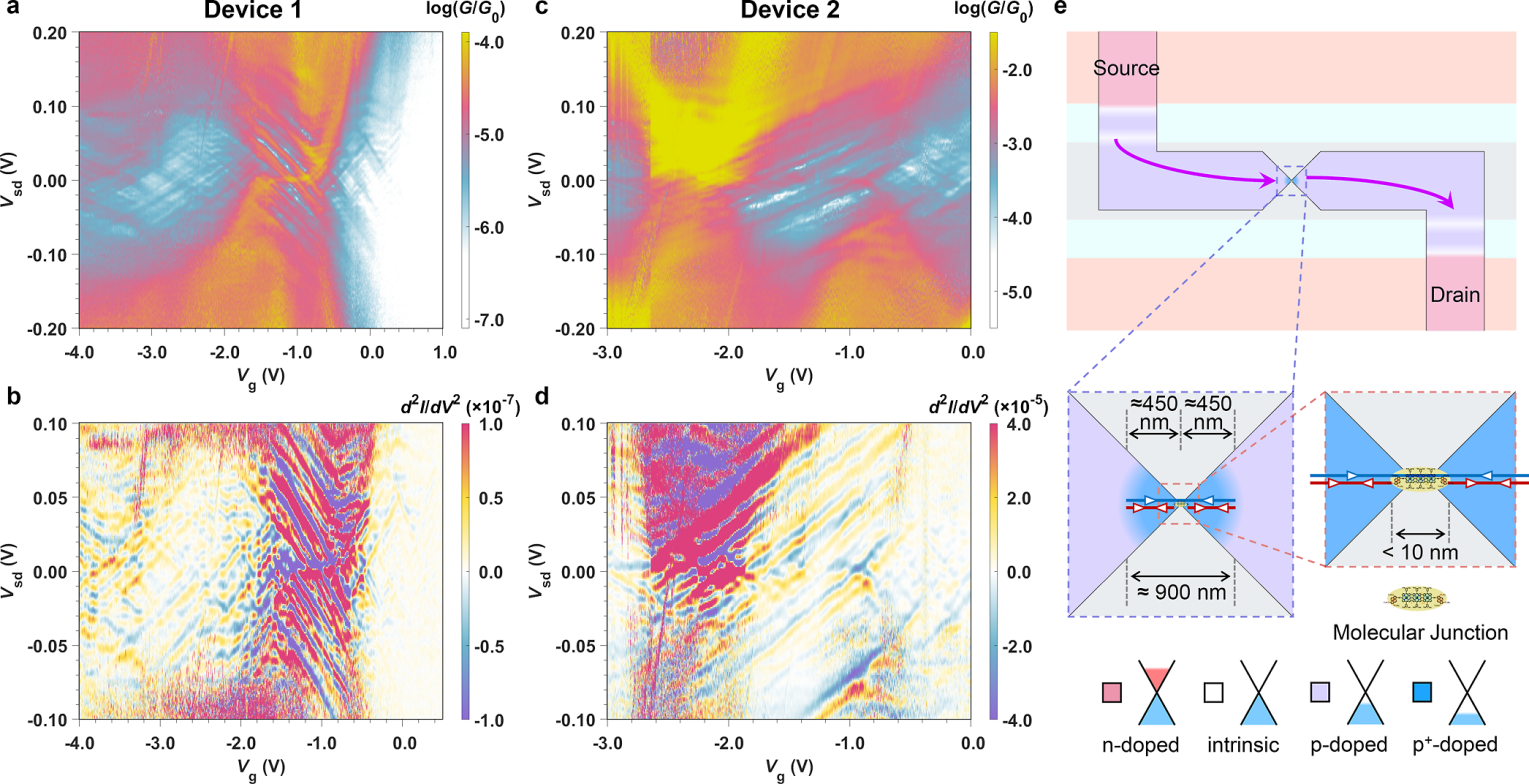
Fabry–Pérot interference. (a,c) Detailed conductance maps of the interference pattern overlapping with molecular charge transitions for **Ni-FP8** device 1 and a second device (device 2), respectively. (b,d) Derivative of differential conductance (d*I*_d_^2^/d*V*_sd_^2^) measured as a function of bias voltage and gate voltage for both devices. (e) Schematic of the possible interference conditions. Two possible resonance conditions for FP interference within the graphene are indicated by arrows within the highly doped region (blue) and on one of the molecule–graphene interfaces (red). As shown in the schematic, the p/p^*+*^ interfaces, generating by Joule heating, may be curved, giving the cavity a confocal or concentric geometry.

A high-resolution conductance map of the *N* – 2*/N* – 1 and *N* – 1*/N* transitions is shown in Figure 2a. There is a periodic structure within the off-resonant conductance, especially in the *V*_sd_ range from −0.1 to +0.1 V, that has a coupling to the gate potential that is weaker than that for the molecular states. As we discuss below, this periodic structure results from electrode states, and the weaker gate coupling is due to a higher carrier concentration in the graphene that more effectively screens the gate electric field.^21^ Furthermore, the coupling depends on the magnitude of the gate voltage itself, ranging from α_g,FP_ = 0.08 eV/V at *V*_g_ ∼ −1 to 0.02 eV/V at *V*_g_ ∼ −4 V (see Supporting Information Figure S4-1). The periodic features are more obvious in the second derivative of the current (d*I*_sd_^2^/d*V*_sd_^2^) map, which displays a “checkerboard” pattern (Figure 2b).

The energy spacings between adjacent conductance oscillations in Figure 2a are ∼4 and ∼8 meV, displaying that the oscillations that make up the checkerboard result from two separate periodicities (shown by FFT analysis and direct calculation in Supporting Information Figures S4-3 and S4-4). Similar periodicities have been observed in transport measurements through graphene and are attributed to the formation of an electronic FP interferometer within the graphene channel.^15,16,39^ By analogy with an optical FP cavity formed from a pair of partially reflective mirrors, two potential steps, induced by doping, define an electronic FP cavity. Considering the resonances within a one-dimensional FP cavity, our measured periodic energy spacings of ∼4 and ∼8 meV correspond to cavity lengths of *L* = *hv*_F_/(2*E*) = 900 and 450 nm, approximating a Fermi velocity of *v*_F_ = 1.8 × 10^6^ m/s by adjusting the value for CVD graphene on SiO_2_ (*v*_F_ = 2.49 × 10^6^ m/s) for the larger dielectric constant of HfO_2_.^40,41^ The length scales of 900 and 450 nm are 2 orders of magnitude larger than those that would be associated with reflections within the **Ni-FP8** nanoribbon itself, confirming that the QI pattern is dominated by reflections within the graphene leads.

The FP cavity is formed within graphene by doping induced by interactions with the underlying substrate. Graphene transferred onto HfO_2_ is p*-*doped^42^ (light purple area shown schematically in Figure 2e). The electroburning process that is used to create the nanogap anneals the graphene local to the constriction.^43^ This effectively cleans the graphene in a region of ∼1000 nm in diameter, as has been observed in AFM and SEM images of electroburnt graphene nanogaps on both SiO_2_^17,39^ and HfO_2_.^44^ Local heating also leads to stronger interactions with the underlying substrate that increase the hole concentration in the area around the tunnel junction, generating a highly doped (p^*+*^-doped) region (blue area in Figure 2e). The change in doping, and cavity length of ∼800–1000 nm, was confirmed by Kelvin probe force microscopy (Supporting Information Figure S1-6).

The interfaces between *p*-doped and *p*^+^-doped graphene regions generate outer potential steps, and the molecule/electrode interfacial tunnel barriers also act as potential steps at which electrons are reflected or transmitted. Therefore, there are two FP cavities formed over ∼450 nm (corresponding to 8 meV energy spacing) that occur between a p/p^*+*^ potential step and a molecule/electrode tunnel barrier on each side of the device. The reflections inside these cavities are indicated by red arrows in Figure 2e. As the length of the molecule is much shorter than the graphene cavity, reflections from either of the interfaces between the molecule and the graphene at their interface with are not distinguishable. The 4 meV energy spacing (900 nm long cavity) indicates an interference process with electron transport back and forth through the partially transmitting molecule,^15^ with reflection occurring on the outer p/p^*+*^ potential steps in the graphene, as indicated by blue arrows in Figure 2e. As the FP resonances extend over the molecular junction, electron transmission through the intermediately coupled molecule must remain phase coherent.

The data for a second, more strongly coupled, **Ni-FP8** device (device 2, Figure 2c,d) and for the two shorter porphyrin oligomer devices (Supporting Information Figure S3-6) also display the same features, with periodic interference patterns that have lower gate couplings than the molecular states. The second **Ni-FP8** device has a stronger molecule–graphene coupling Γ than device 1, increasing the conductance, and consistent with a weaker coupling of the more hybridized molecular states to the gate potential (lower α_g,mol_). All four devices share the property of having stronger conductance fluctuations when the molecule is oxidized to the *N* – 1 state (also shown in the *G*–*V*_g_ trace in Figure 1d), which may be related to the molecule being more transmissive upon oxidation (discussed in more detail below). For device 1, the nanoribbon is more strongly coupled to the source electrode (i.e., Γ_S_ > Γ_D_), which can be inferred from the regions of resonant transport that are higher in conductance,^28^ whereas the opposite is true for device 2 (Γ_D_ > Γ_S_).

### Phase-Coherent Molecular Transport

**Figure 3:**
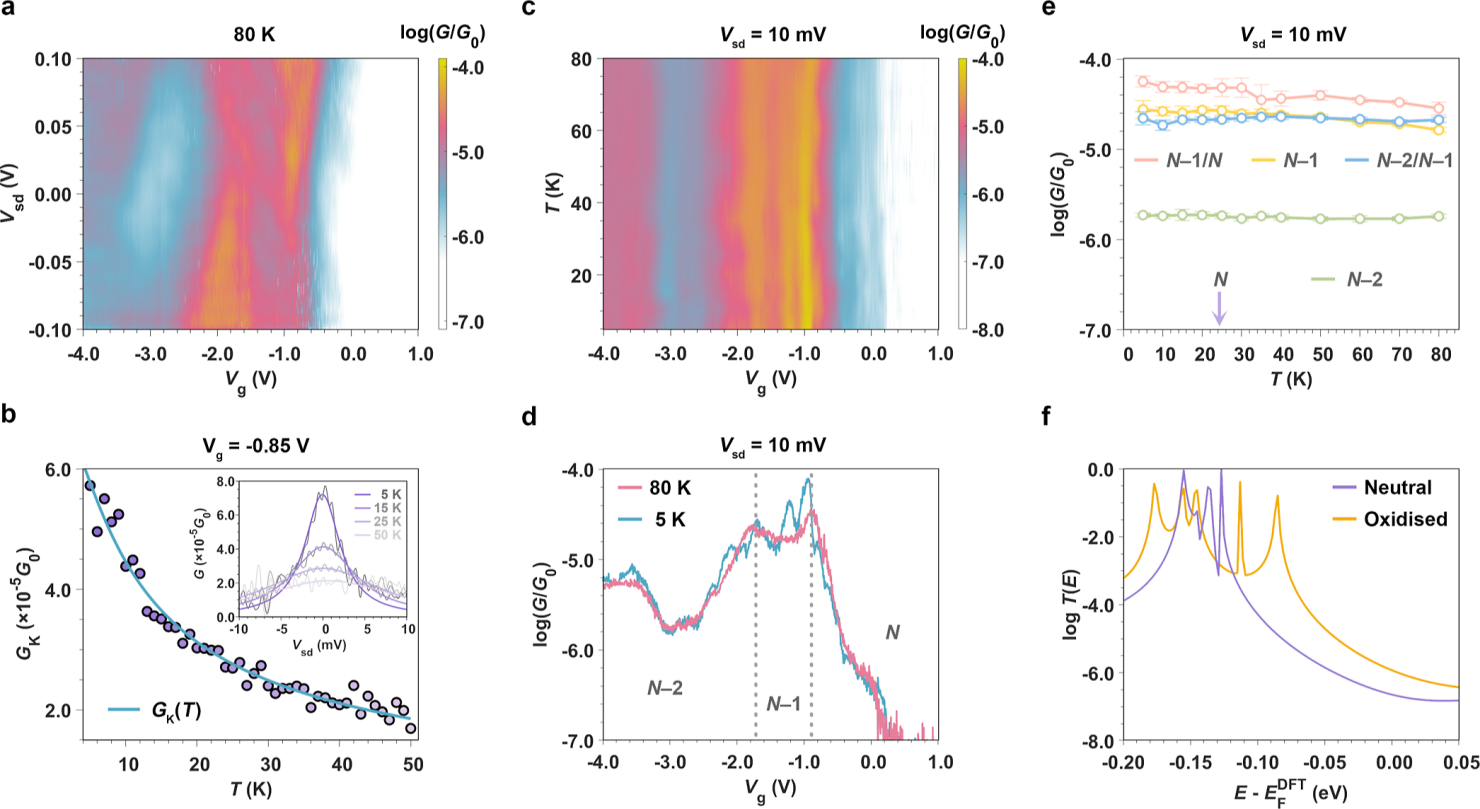
Temperature and oxidation-state dependence of molecular conductance. (a) Fluctuations of conductance and Kondo resonances from interference disappear as thermal energy surpasses *hv*_F_/*L* (4 meV) and the coherence length decreases, as shown in the conductance map at 80 K (*k*_B_*T* = 6.9 meV). (b) Experimental differential conductance of the Kondo peak at *V*_g_ = −0.85 V (purple circles) and fit to the spin-1/2 Kondo model with *T*_K_ = 18 ± 1 K and *s* = 0.30 ± 0.04 (blue line). Inset: Experimental differential conductance as a function of bias voltage (gray curves) and the Lorentzian fits of Kondo peaks (purple curves). (c) Differential conductance map measured as a function of temperature and gate voltage. Indicative of phase coherent transport, the conductance does not show an obvious temperature dependence. (d) Differential conductance as a function of *V*_g_ measured at 5 K (blue curve) and 80 K (pink curve) with a fixed bias voltage *V*_sd_ = 10 mV. (e) Temperature dependence of off-resonant transport measured in the conductance minima of the charge states. The values are averaged over a 100 mV window around *V*_g_ = −2.90 V for *N* – 2, *V*_g_ = −1.30 V for *N* – 1, and *V*_g_ = 0.6 V for *N* charge states. We also display the temperature dependence of the *N* – 2/*N* – 1 (*V*_g_ = −1.75 V) and *N* – 1/*N* (*V*_g_ = −0.95 V) Coulomb peaks. Note that there is a shift in the position of the Coulomb peaks from Figure 2, probably as a result of a change in trap occupancy in the HfO_2_. For (c–e), the conductance was measured at *V*_sd_ = 10 mV to exclude the impact of Kondo resonance. (f) Calculated transmission spectra for the neutral state (purple) and oxidized state (orange) of a single-molecule junction. All experimental data in this figure are from **Ni-FP8** device 1.

Next, we describe the temperature and charge-state dependence of device conductance in more depth. As shown in Figure 3a, as the thermal energy increases beyond *hv*_F_/*L* (4 meV), the FP interference pattern disappears, probably because the thermal energy becomes larger than the FP spacing (at 80 K, *k*_B_*T* = 6.9 meV), and the coherence length within the graphene channel decreases with increasing temperature.^45^ Furthermore, the Kondo resonance, clearly visible as a zero-bias conductance enhancement within the *N* – 1 state (**Ni-FP8**^**+**^) at 5 K, as shown in Figure 2a, is also no longer present in the 80 K data. The Kondo resonance results from scattering from a many-body state formed between a molecular spin and electrons of opposite spin at the Fermi level of the electrodes. When **Ni-FP8** is oxidized to the *N* – 1 state (**Ni-FP8**^**+**^), an electron is removed from the molecular π-system and the resulting spin (*S* = 1/2) and charge are delocalized over the molecule, forming the Kondo state with electrons in the graphene electrodes. As thermal energy increases toward the binding energy of this many-body molecule-electrode state, the zero-bias conductance will decay. The characteristic temperature-dependence is parameterized by the Kondo temperature (*T*_K_), the temperature when the conductance is half its value at 0 K, i.e., *G*(*T* = *T*_K_) = 0.5 × *G*(*T* = 0). We extract the Kondo temperature of our device at *V*_g_ = −0.85 V by fitting the temperature-dependence of the zero-bias conductance to the usual spin-1/2 model
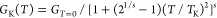
where *s* is an empirical parameter.^46^

We calculate *T*_K_ = 18 ± 1 K and *s* = 0.30 ± 0.04 from the fit in Figure 3b. We also obtain *T*_K_ at this gate voltage from the fwhm of the resonance, which gives a similar value of 17 ± 1 K (see Figure 3b, inset). There are several parameters that define Kondo temperature of a nanoscale system; it depends on the addition energy of the *N* – 1 transition (0.12 eV for **Ni-FP8** device 1) and exponentially on the molecule–electrode coupling, hence its association with the onset of intermediate coupling.^10^ A typical gate-dependent measurement of a Kondo resonance would give a smooth conductance decay (or Kondo valley) as *V*_g_ is detuned from the resonances, described by the Haldane relation.^34,46^ However, as *T*_K_ also depends on the graphene density of states,^47^ our gate-dependent measurement of the Kondo peak displays conductance oscillations, as opposed to a smooth valley, within the *N* – 1 state, in line with a previous observation of transmission through a coupled Kondo/FP system.^48^ The Kondo resonance is not visible in the *N* + 1 state, probably because the gate voltages in which the molecule is in the singly reduced state are close to the Dirac point of graphene (at *V*_g_ = +1.6 V, see Supporting Information Figure S4-1), or alternatively a weaker molecule–electronic coupling to that charge state due to transport through more localized orbitals (see Supporting Information Figure S5-5), or a combination of both. Aside from the Kondo behavior, the conductance of the device, including at the Coulomb peaks, show a weak temperature dependence (Figure 3c,d). In the regions of *V*_g_ between the Coulomb peaks, the conductance is temperature independent, with some slight decrease in the conductance of the *N* – 1 state and the *N* – 1/*N* peak due to Fermi broadening and consistent with off-resonant phase-coherent transport being the dominant mechanism (more temperature-dependent data are provided in Supporting Information Section S3).

The porphyrin nanoribbon **Ni-FP8** constitutes one of the longest molecular systems (8 nm) with well-defined anchor groups over which phase-coherent transport has been measured, consistent with previous conductance measurements that demonstrate low or negative attenuation factors for edge-fused porphyrin oligomers.^24,49^ This is a property of the molecule and results from strong coupling between the fused porphyrin units and subsequent delocalization of electronic wavefunctions over the whole π-system, as corroborated by measurements of the optical gap.^50^ Another molecular-structure-dependent charge transport property is the presence of the 2 Coulomb peaks (*N* – 1/*N* and *N* – 1/*N* – 2) at negative *V*_g_ that are due to oxidation of the electron-rich TDP anchor groups, coupled through the porphyrin nanoribbon.^51^ Thus, the two **Ni-FP8** devices share this relatively similar feature with **Zn-P1** and **Zn-FP3** (Supporting Information Figure S3-6), with a different addition energy dependent on the length of the fused porphyrin core.

It is striking that the off-resonance conductance and the appearance of the phase-coherent FP interference pattern is strongly dependent on the charge state of **Ni-FP8,** as well as depending on *V*_g_ within each diamond due to the effect of the gate potential on the level alignments (Figure 3d). For the neutral nanoribbon, the conductance is below the noise level (<10^–7.0^*G*_0_) of our experimental setup away from the Coulomb peaks, but it is 10^–4.7^*G*_0_ in the mid-gap of the *N* – 1 state and 10^–5.8^*G*_0_ in *N* – 2, indicating that in the oxidized states of the **Ni-FP8**, off-resonance coherent transport is more efficient than in the neutral state. The same trend is observed in **Ni-FP8** device 2, but with higher conductance values due to stronger molecule–electrode coupling, with values of 10^–3.6^*G*_0_, 10^–3.2^*G*_0_, and 10^–3.4^*G*_0_ for *N*, *N* – 1, and *N* – 2 charge states, respectively. The difference in magnitudes between device 1 and device 2 highlights the continuing need for strategies to be developed to control Γ so that devices with specific properties (rather than trends) are able to be fabricated on a larger scale.

Charge-state-dependent conductance has been observed previously in STM-BJ measurements of fused porphyrin oligomers,^52^ with a similar conductance enhancement of ∼100 reported for an edge-fused trimer upon oxidation, similar to the ratio measured in our device that has a quite different geometry. Furthermore, our results are consistent with the general observation in single-molecule conductance measurements that upon oxidation or reduction, a conductance enhancement is observed in the odd-electron number state.^53−55^ As with these previous studies, we can utilize a combination of DFT and quantum transport theory^56−58^ to calculate the phase-coherent transmission (see the details in the Experimental Section) to support our experimental observations of a change in off-resonant conductance after **Ni-FP8** oxidation. In order to calculate the transmission of the fused octamer in the oxidized state, a theoretical model was constructed with chlorine atoms placed on top of each nickel atom in the fused octamer. The Cl atoms mimic the effect of the gate potential in that they cause the net transfer of an electron from the molecule, thereby enabling transmission to be calculated for an oxidized nanoribbon. The transmission coefficient *T*(E) (orange curve), when eight Cl atoms are present, is plotted in Figure 3f, and for comparison, the transmission coefficient (purple curve) of the neutral octamer is also shown. In agreement with our measurements and even at finite bias (i.e., above the Kondo resonance that arises from many-body effects not captured within the calculations), a clear increase in *T*(*E*) is observed over a large energy range close to Fermi energy upon oxidation, primarily due to a shrinking of the HOMO–LUMO gap (Supporting Information Figure S5-2). As shown in Supporting Information Figure S5-4, the increase in transmission upon oxidation is robust to changes in the portion of the nanoribbon overlapping with each of the graphene electrodes. The introduction of Cl atoms creates extra features (spikes) in the transmission that does not affect the increasing trend (Supporting Information Figure S5-2). The calculated transmission spectrum based on coherent transport theory shows excellent agreement with the experimental measurements of **Ni-FP8**, particularly at higher temperature.

## Discussion

Our results show that phase-coherent electron transport persists through a fused porphyrin nanoribbon even over a molecular length of 8 nm. The mechanism is confirmed by measurement of charge-state-dependent interference fringes from FP resonances that extend over a graphene–molecule–graphene cavity, along with the observation of temperature-independent off-resonant conductance in multiple oxidation states of the molecule. The persistence of phase-coherent transport behavior across such extended molecular systems within the intermediate molecule–electrode coupling regime is important for understanding and designing systems for efficient long-range electron transport. The gate electrode can be used to precisely tune the thermodynamic driving-force of electron-transfer reactions, and therefore this platform could be extended to study long-range electron transmission and transfer in biological systems appropriately functionalized to interface with graphene.^59,60^

The study of coherent electron transport through a molecular nanoribbon embedded in a FP cavity opens up prospects for all-electrical interferometric measurements between graphene and molecular pathways, where both transmission magnitude and phase through the device can be determined experimentally. This platform could enable the readout of molecular topological qubits, for which nanoribbons have demonstrated potential,^4^ therefore providing an interesting research direction in exploring nanoribbon–graphene hybrid devices for quantum information processing.

## Experimental Section

### Synthesis of Ni-FP8

To a solution of **Ni-LP8Br** (2.0 mg, 0.15 μmol, 1 equiv) in dry 1,2-dichloroethane (DCE, 2.5 mL), a suspension of AuCl_3_ (0.64 mg, 2.1 μmol, 14 equiv) and AgOTf (2.7 mg, 11 μmol, 70 equiv) in dry DCE (2.5 mL) was added dropwise, and the reaction mixture was stirred at 25 °C for 15 min. After that, a suspension of AuCl_3_ (0.13 mg, 0.42 μmol, 2.8 equiv) and AgOTf (0.54 mg, 2.1 μmol, 14 equiv) in dry DCE (0.5 mL) was added dropwise to the reaction mixture, and the reaction was monitored by UV–vis–NIR spectroscopy with CH_2_Cl_2_ + 1% triethylamine as the solvent. After completion, triethylamine (1.0 mL) was added to the reaction mixture. The resulting mixture was purified by flash column chromatography on silica gel using pentane/CH_2_Cl_2_ (9:1) as the eluent to give product **Ni-FP8Br** (1.0 mg, 50% yield).

A mixture of **Ni-FP8Br** (1.0 mg, 0.075 μmol, 1.0 equiv), Pd(PPh_3_)_4_ (1.1 mg, 1.5 μmol, 20 equiv), and CuI (0.14 mg, 0.75 μmol, 10 equiv) in dry toluene (0.5 mL) and diisopropylamine (DIPA, 0.5 mL) was degassed by three freeze–pump–thaw cycles. A solution of 1,3,6-tris(dodecyloxy)-8-ethynylpyrene (10 mg, 13 μmol, 170 equiv) in dry toluene (0.5 mL) and DIPA (0.5 mL) was degassed by three freeze–pump–thaw cycles and transferred to the reaction mixture under argon. After that, the mixture was stirred at 50 °C under argon for 2 h. Then, a degassed solution of 1,3,6-tris(dodecyloxy)-8-ethynylpyrene (5.0 mg, 6.4 μmol, 85 equiv) in dry toluene (0.5 mL) and DIPA (0.5 mL) was added to the reaction mixture and the mixture was stirred at 50 °C for another 20 h. After reaction, the resulting mixture was separated by flash column chromatography on silica gel using pentane/CH_2_Cl_2_ (1:1) as eluent, followed by size-exclusion chromatography (Biorad Bio beads SX-1) with toluene/pyridine (99:1) as the eluent to give the crude mixture. The crude mixture was further subjected to recycling GPC with toluene/pyridine (99:1) as the eluent to separate the desired product **Ni-FP8** (0.11 mg, 11%). See Supporting Information Section S2 for the reactions and Supporting Information Section S2 for characterization data of intermediate compounds.

### Substrate Fabrication

The substrate used for **Ni-FP8** devices was fabricated using the following procedure. On a degenerately n-doped silicon wafer with a layer (300 nm thick) of thermally grown silicon dioxide (SiO_2_), a local gate electrode (3 μm wide) was defined by optical lithography with lift-off resist and electron-beam (e-beam) evaporation of titanium (5 nm thick) and platinum (15 nm thick). A layer (10 nm) of hafnium dioxide (HfO_2_) was then deposited using atomic layer deposition (ALD). Next, source and drain contact electrodes separated by a 7 μm gap (the center of the gap was aligned to the center of the gate electrode, which means a 2 μm of horizontal distance between each electrode and gate electrode) were also defined by optical lithography with lift-off resist and e-beam evaporation of titanium (5 nm thick) and platinum (45 nm thick). The procedure for the fabrication of substrates used for **Zn-P1** and **Zn-FP3** has been published previously.^44^

### Graphene Nanogaps

A layer (600 nm) of poly(methyl methacrylate) (PMMA) (with a molecular weight of 495 kDa) was spin-coated onto chemical vapor deposition (CVD)-grown graphene (purchased from Grolltex) on copper. The copper was then etched in aqueous ammonium persulfate ((NH_4_)_2_S_2_O_8_) solution (3.6 g in 60 mL water) for 4 h, after which the PMMA-protected graphene was transferred three times to Milli-Q water and scooped up using the substrate. Air bubbles were removed by partly submerging the sample in 2-propanol (IPA). The sample was dried overnight and baked at 180 °C for 1 h. The PMMA was then removed in hot acetone (50 °C) for 3 h.

The Z-shaped graphene tape with bow-tie shaped structure was patterned by e-beam lithography (EBL) with a bi-layer lift-off resist (PMMA495 and PMMA950) and thermal evaporation of aluminum (50 nm thick). The Z-shaped graphene pattern was used so the inner graphene leads are coplanar with the bowtie structure (see Figure 1b,c), reducing tension on the bowtie-shaped graphene and maximizing the stability of the junction. A PMMA e-beam resist was used as it is a positive resist and it is transformed into smaller molecules after exposure, which make it much easier to be removed than a negative photoresist. Aluminum was then deposited onto exposed areas as an oxygen-plasma resist, as aluminum can be completely removed by either acidic or basic aqueous solutions. By this method, we reduce contamination from residual photoresist on graphene. The flatter configuration and cleaner surface might provide stronger molecule–electrode coupling by better molecule–graphene interfacing. After liftoff, the graphene on unexposed areas (which are not covered by aluminum) was etched with oxygen plasma. The aluminum was subsequently removed by aqueous sodium hydroxide (NaOH) solution (0.5 M; 1.0 g in 50 mL water). The sample was finally immersed in hot acetone (50 °C) overnight to remove any residual PMMA. The optical image and SEM images can be found in Supporting Information Section S1.

Graphene nanogaps were prepared by feedback-controlled electroburning of the graphene bow-tie shape until the resistance of the tunnel junction exceeds 1.3 GΩ (10^–7^*G*_0_). The empty nanogaps were characterized by measuring a current map as a function of bias voltage (*V*_sd_) and gate voltage (*V*_g_) at room temperature in order to exclude devices containing residual graphene quantum dots;^17^ only clean devices were selected for further measurement.

### Molecule Junctions and Measurements

The solution of the porphyrin nanoribbon (1 μM in toluene) was drop-cast on electroburnt graphene electrodes and allowed to dry in air. Only devices that showed clean current maps before molecule deposition were selected for further measurements. Thus, new signals appearing after molecule deposition can be attributed to transport through molecular junctions. Then, the chip containing molecular devices was connected to a chip holder via wire bonding, loaded in Oxford Instruments 4K PuckTester, and cooled down to cryogenic temperature for detailed measurements. The current maps and differential conductance maps of before and after measurements can be found in Supporting Information Section S3.

### Theoretical Calculations

Geometrical optimizations were carried out using the DFT code SIESTA,^56^ with a local density approximation LDA functional, a double-ζ polarized basis, a cutoff energy of 200 Ry, and a 0.04 eV/Å force tolerance. From the Hamiltonian and overlap matrices of the DFT calculation of the junction, Gollum^57^ calculates the transmission coefficient *T*_nm_(*E*) between scattering channels n and m in the electrodes, from which the transmission coefficient  is obtained. As discussed in chapter 17 of ref (58), this is equivalent to the expression
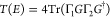
where *G* is the (retarded) Green’s function of the junction and Γ_*i*_ is the imaginary part of the self-energy of electrode *i*. The electrical conductance is obtained from
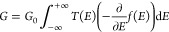
where *E*_F_ is the Fermi energy of the device,  is the Fermi distribution function, and  is the conductance quantum. At low enough temperatures, this is approximated by *G* = *G*_0_*T*(*E*_F_). In the presence of Cl atoms, spin-polarized calculations were carried out to obtain the transmission coefficients *T*↑, *T*↓ for the two different spins, from which the total transmission coefficient  is obtained.

## Supporting Information Available

Details of fabrication, nanoribbon synthesis, transport measurement, data analysis, and DFT calculation (PDF)
