# Supplementary material for: Phase-Coherent Charge Transport through a Porphyrin Nanoribbon
Source: J Am Chem Soc. 2023 Jul 7;145(28):15265–74. doi: 10.1021/jacs.3c02451 (PMC10360058; doi:10.1021/jacs.3c02451)
Supplement: Supplementary file 1 — ja3c02451_si_001.pdf [file ja3c02451_si_001.pdf]

Supporting Information:

Phase-Coherent Charge Transport through a Porphyrin Nanoribbon

Zhixin Chen[1,7,*], Jie-Ren Deng[2,7], Songjun Hou[3], Xinya Bian[1], Jacob L. Swett[1], Qingqing Wu[3], Jonathan Baugh[4], Lapo Bogani[1], G. Andrew D. Briggs[1], Jan A. Mol[5], Colin J. Lambert[3,*], Harry L. Anderson[2,*], and James O. Thomas[1,6,*]

[1]Department of Materials, University of Oxford, Parks Road, Oxford, OX1 3PH, UK

[2]Department of Chemistry, University of Oxford, Chemistry Research Laboratory, Oxford, OX1 3TA, UK

[3]Department of Physics, Lancaster University, Lancaster, LA1 4YB, UK

[4]Institute for Quantum Computing, University of Waterloo, Waterloo, ON N2L 3G1, Canada

[5]School of Physical and Chemical Sciences, Queen Mary University, London, E1 4NS, UK

[6]Lead contact

[7]These authors contributed equally

*Correspondence: *zhixin.chen@materials.ox.ac.uk (Z. C.), *c.lambert@lancaster.ac.uk (C. J. L.), *harry.anderson@chem.ox.ac.uk (H. L. A.), *james.thomas@materials.ox.ac.uk (J. O. T.)

Contents

Supplemental Section 1	Fabrication	2
Supplemental Section 2	Nanoribbon Synthesis	5
Supplemental Section 3	Transport Measurement	15
Supplemental Section 4	Data Analysis	20
Supplemental Section 5	DFT Calculation	23
Supplemental References		26

Supplemental Section 1 Fabrication

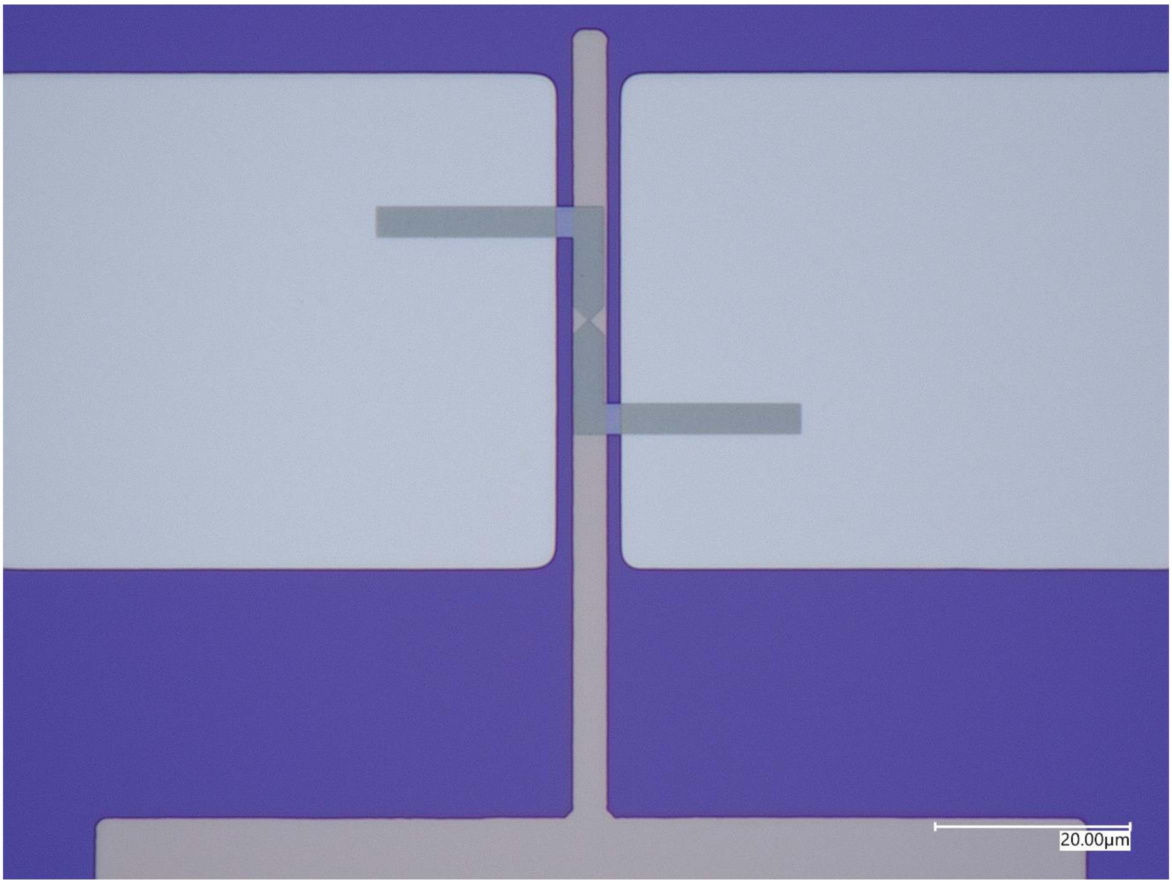

Fig. S1-1. Example optical image of the Al protected device, fabricated as described in the Methods section. The Z-shaped graphene tape with a bow-tie center was protected by a 50 nm thick layer of aluminum.

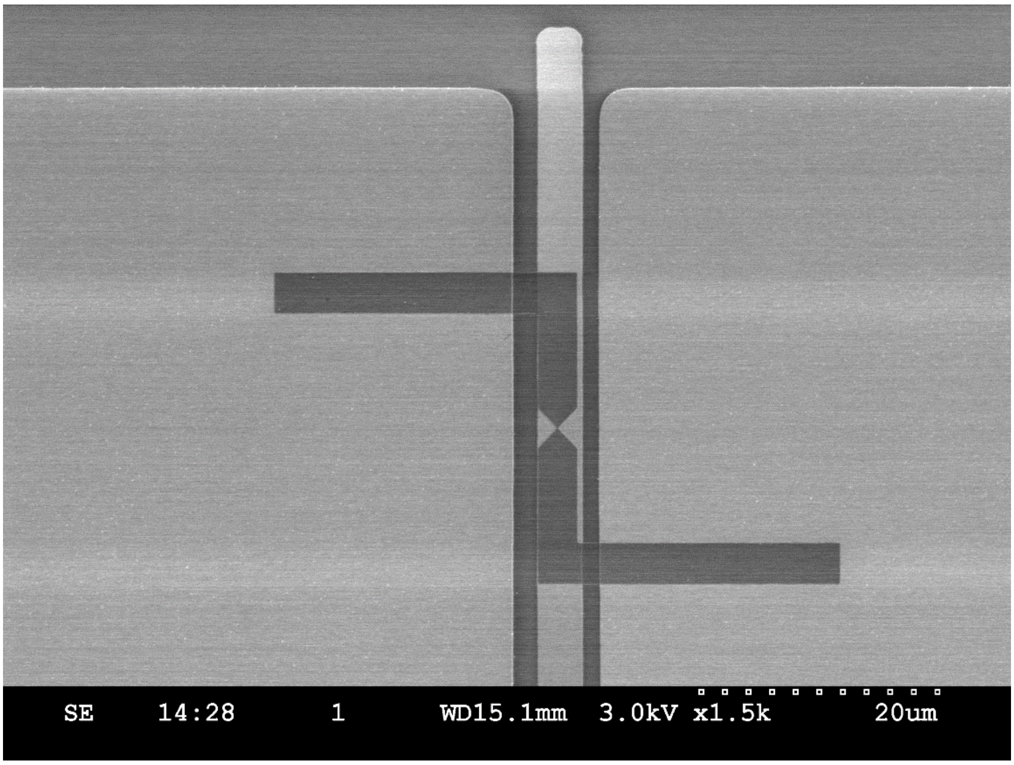

Fig. S1-2. Example SEM image of the aluminum-protected device.

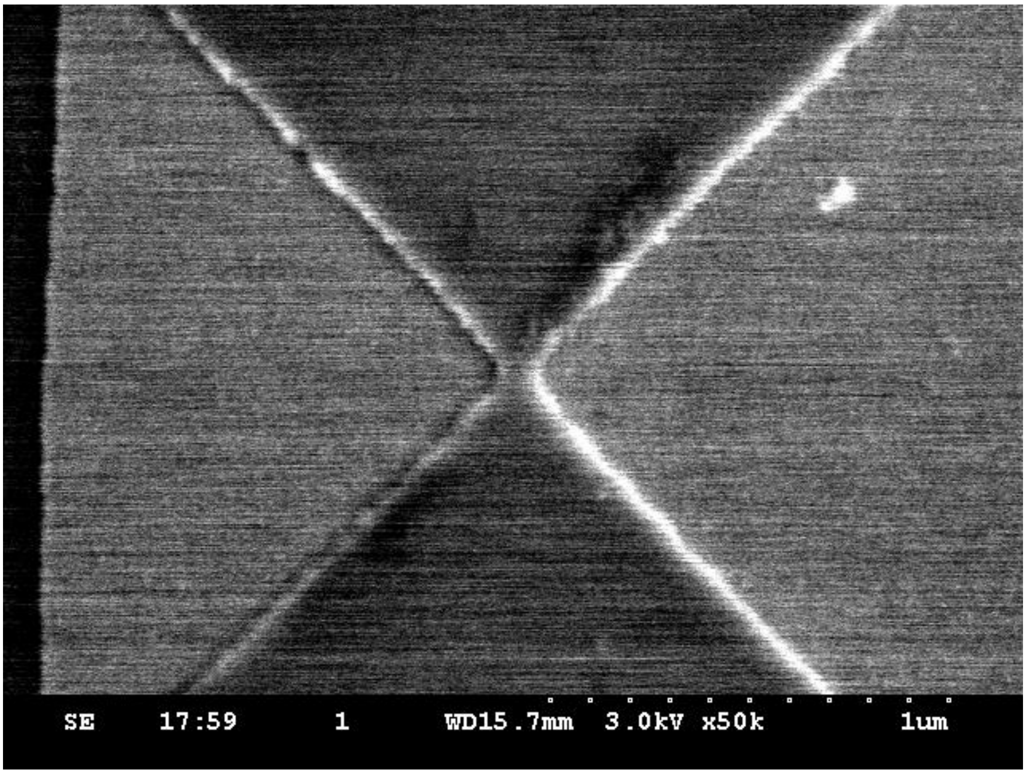

Fig. S1-3. Example SEM image of the aluminum protected device (zoom-in view of the bow-tie shaped structure).

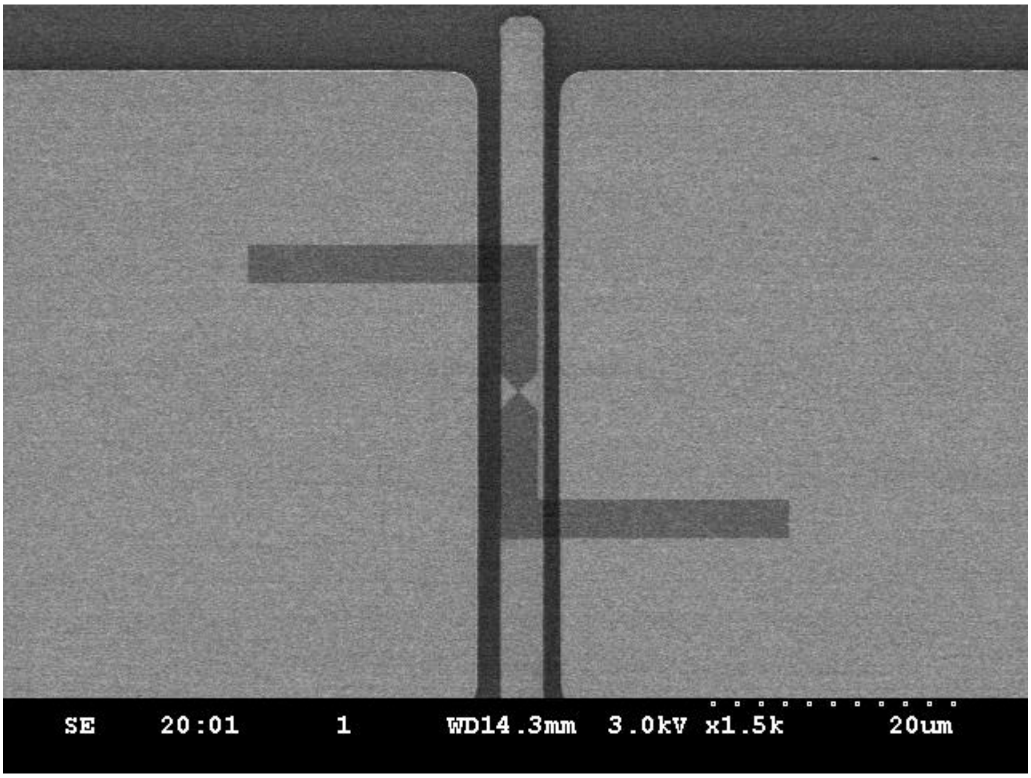

Fig. S1-4. Example SEM image of the device (the aluminum and residual PMMA have been removed).

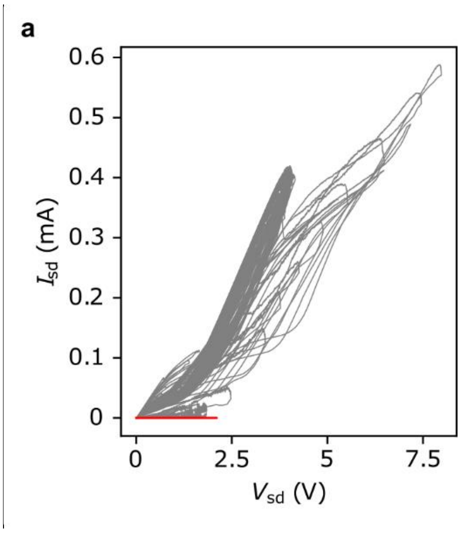

Fig. S1-5. Feedback-controlled electroburning traces used to produce the graphene nanogap of device 1.

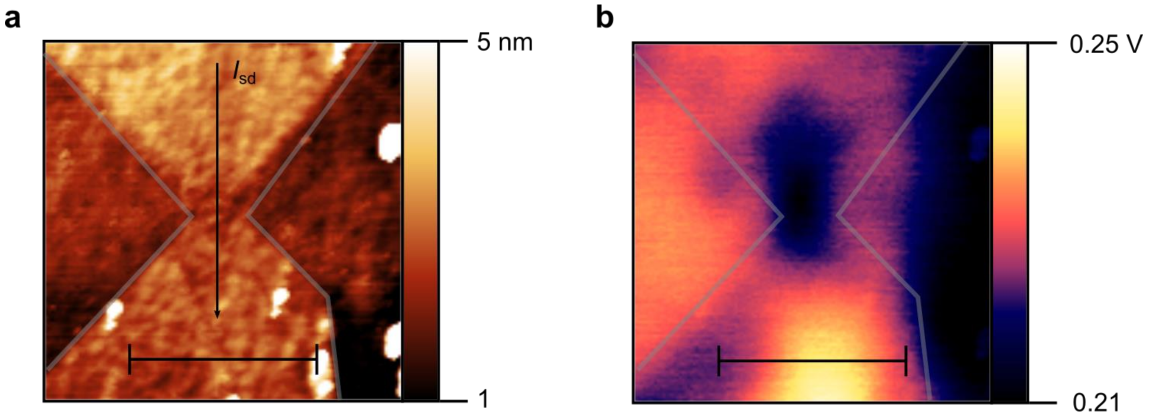

Fig. S1-6. (**a**) Topographic and (**b**) contact potential difference (CPD) measurements of a graphene constriction after electoburning using Kelvin probe force microscopy. The region of graphene annealed during electroburning is ~ 800–1000 nm in length, and visible as a p+ doped (dark) region in the CPD map. This is consistent with the cavity length calculated from ~ 4 meV energy spacing with Fermi velocity v_F = 1.8 × 10^6 m/s. The size is also consistent with previous reports of Joule-heated graphene constrictions on SiO_2[1,2] and HfO_2,[3] where the annealed region could be identified as cleaner than the surrounding graphene in a topographic image, but the novel fabrication procedure used here (see Results and Experimental section) leads to less residual photoresist contamination and this effect is not present. The grey outlines define the graphene channel, which has partly folded in the lower right hand side. The direction of the source-drain current is indicated by the arrow. Scale bars are 1 μm.

Supplemental Section 2 Nanoribbon Synthesis

General Procedures

All reagents were purchased as reagent grade and used without further purification. Solvents for column chromatography were HPLC grade. Dry toluene and diisopropylamine (DIPA) were obtained from the solvent drying system MBraun MBSPS-5-BenchTop under nitrogen atmosphere (H_2O content < 20 ppm as determined by Karl-Fischer titration). Flash column chromatography was carried out using SiO_2 (60 Å, 230–400 mesh) under positive pressure. Analytical thin-layer chromatography was carried out on aluminum-backed silica gel 60 F254 plate. Evaporation in vacuum was performed at 25–80 °C and 900–10 mbar. Size exclusion chromatography (SEC) was carried out using Bio-Rad Bio-Beads S-X1 (40–80 µm bead size). Semi-preparative gel permeation chromatography (GPC) was carried out on a Shimadzu recycling GPC system equipped with a LC-20 AD pump, SPD-20A UV detector and a set either of JAIGEL 3H (20 × 600 mm) and JAIGEL 4H (20 × 600 mm) columns using toluene/pyridine (99:1) as eluent at a flow rate of 3.5 mL/min. Reported yields refer to spectroscopically and chromatographically pure compounds that were dried under high vacuum (0.01–0.005 mbar) before analytical characterization.

NMR spectra were recorded at 298 K using a Bruker NEO 600 with a broadband helium cryoprobe, or a Bruker AVIII 700 with an inverse TCI $^{1}H/^{13}C/^{15}N$ cryoprobe. ^{1}H NMR spectrum is reported in ppm; coupling constants are given in Hertz, to the nearest 0.1 Hz. The solvent used was $CDCl_3$ which was calibrated to residual $CHCl_3$ at 7.26 ppm. Multiplicity (s = singlet, d = doublet, t = triplet, q = quartet, m = multiplet) and coupling constants were reported whenever possible. ^{1}H NMR signals were assigned based on comparison between compounds, chemical shifts, integrals and coupling constants.

MALDI-ToF spectra were measured using a Bruker MALDI Autoflex Speed spectrometer utilizing *trans*-2-[3-(4-*tert*-butylphenyl)-2-methyl-2-propenylidene]-malononitrile (DCTB) as matrix.

UV-vis-NIR absorbance measurement was recorded with a Perkin-Elmer Lambda 20 spectrophotometer or a Jasco V770 spectrophotometer using an Infrasil® Quartz 1 cm cuvette. Measurements were carried out at 25 °C under ambient conditions.

Synthesis and Characterization of *l*-ZnP8$_H$

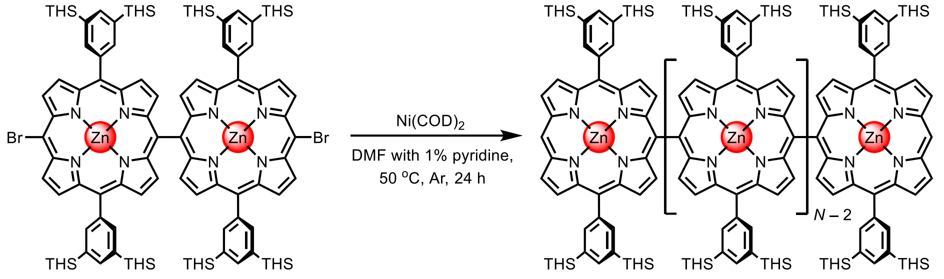

***l*-ZnP2$_{Br}$** ***l*-ZnP*N*$_H$**

A mixture of *meso*–*meso* singly-linked zinc(II) porphyrin dimer ***l*-ZnP2$_{Br}$**[1] (500 mg, 144 μmol, 1.0 equiv.), Ni(COD)$_2$ (159 mg, 577 μmol, 4.0 equiv.) in dry DMF (150 mL) with pyridine (1.5 mL) was evacuated and backfilled with argon for five times. The reaction mixture was stirred at 50 °C under argon for 24 h. After reaction, the mixture was concentrated, dissolved in pentane/CH_2Cl_2 (19:1), purified by flash column chromatography (silica gel) using pentane/CH_2Cl_2 (9:1) as eluent, followed by size-exclusion chromatography (Bio-Beads, S-X1) with toluene/pyridine (99:1) as eluent to give the crude mixture, which contained a series of *meso*–*meso* singly-linked zinc(II) porphyrin oligomers with different number of porphyrin units. The crude mixture was further subjected to recycling GPC with toluene/pyridine (99:1) as eluent to separate the products ***l*-ZnP2$_H$** (17 mg, 3.5% yield), ***l*-ZnP4$_H$** (23 mg, 4.8% yield), ***l*-ZnP6$_H$** (24 mg, 5.1% yield), ***l*-ZnP8$_H$** (56 mg, 12% yield), ***l*-ZnP10$_H$** (27 mg, 5.6% yield), ***l*-ZnP12$_H$** (23 mg, 4.9% yield) and ***l*-ZnP14$_H$** (12 mg, 2.6% yield).

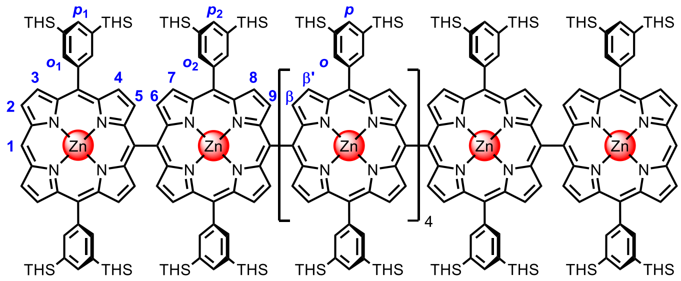

^{1}H NMR (600 MHz, CDCl$_3$, 298 K): δ 10.44 (s, 2H, ***H$_1$***), 9.52 (d, J = 4.4 Hz, 4H, ***H$_2$***), 9.15 (d, J = 4.4 Hz, 4H, ***H$_3$***), 8.84–8.74 (m, 24H, ***H$_{4,7/8,β'}$***), 8.69 (d, J = 4.6 Hz, 4H, ***H$_{7/8}$***), 8.39–8.30 (m, 56H, ***H$_{5,6/9,β,o1,o2,o}$***), 8.18 (d, J = 4.6 Hz, 4H, ***H$_{6/9}$***), 7.95 (s, 4H, ***H$_{p1}$***), 7.89–7.83 (m, 8H, ***H$_p$***), 7.81 (s, 4H, ***H$_{p2}$***), 1.46–0.32 (m, 1184H, ***H$_{THS}$***).
MALDI-ToF: m/z = 13237.28 ($C_{832}H_{1362}N_{32}Si_{32}Zn_8$, $M^{•+}$ requires 13235.46).
UV-vis-NIR (CDCl$_3$, 298 K) λ$_{max}$ (ε / 10^5 M^{-1} cm^{-1}): 412 (7.73), 497 (7.02), 575 (4.08) nm.

Synthesis and Characterization of *l*-ZnP8$_{Br}$

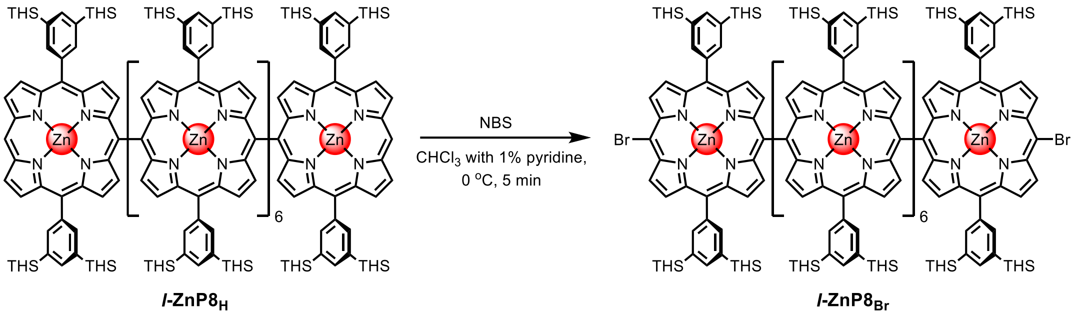

To a solution of ***l*-ZnP8$_H$** (16 mg, 1.2 µmol, 1.0 equiv.) in $CHCl_3$ (5.0 mL) with pyridine (50 µL) in an ice bath (0 ºC), a solution of *N*-bromosuccinimide (NBS, 0.45 mg, 2.5 µmol, 2.1 equiv.) in $CHCl_3$ (1.0 mL) was added dropwise. The reaction was conducted at 0 ºC for 5 min, and acetone (0.1 mL) was added to quench the excess *N*-bromosuccinimide. The resulting mixture was purified by flash column chromatography on silica gel using pentane/CH_2Cl_2 (9:1) as eluent to give the desired product ***l*-ZnP8$_{Br}$** (13 mg, 82% yield).

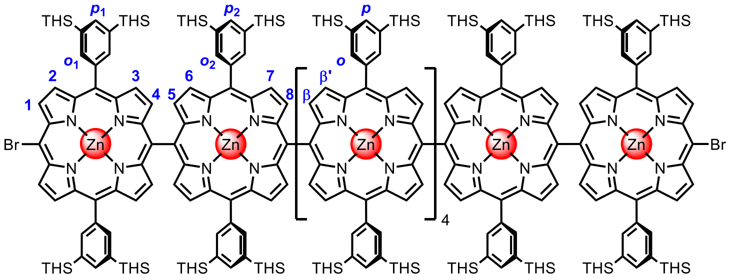

^{1}H NMR (600 MHz, CDCl$_3$, 298 K): δ 9.89 (d, *J* = 4.6 Hz, 4H, H_1), 9.04 (d, *J* = 4.6 Hz, 4H, H_2), 8.82 – 8.74 (m, 20H, $H_{6/7,\beta'}$), 8.73–8.68 (m, 8H, $H_{3,6/7}$), 8.40–8.29 (m, 52H, $H_{5/8,\beta,o1,o2,o}$), 8.24 (d, *J* = 4.6 Hz, 4H, H_4), 8.18 (d, *J* = 4.6 Hz, 4H, $H_{5/8}$), 7.94 (s, 4H, H_{p1}), 7.87–7.83 (m, 8H, H_p), 7.82 (s, 4H, H_{p2}), 1.46–0.41 (m, 1184H).
MALDI-ToF: *m*/*z* = 13394.90 ($C_{832}H_{1360}Br_2N_{32}Si_{32}Zn_8$, $M^{\bullet+}$ requires 13393.28).
UV-vis-NIR (CDCl$_3$, 298 K) λ_{max} (ε / 10^5 M^{-1} cm^{-1}): 416 (8.90), 497 (8.90), 576 (5.13) nm.

Synthesis and Characterization of *l*-H_2P8_{Br}

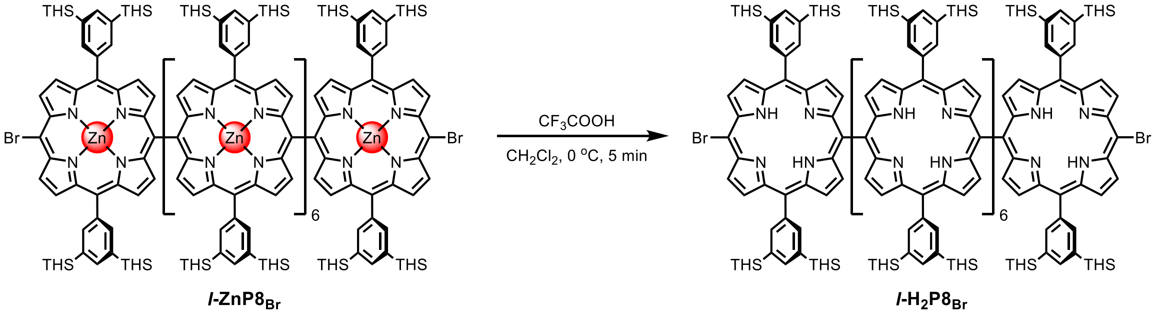

To a solution of ***l*-ZnP8**$_{Br}$ (8.3 mg, 0.62 μmol) in CH_2Cl_2 (2.5 mL) in an ice bath (0 °C), a solution of CF_3COOH (100 μL) in CH_2Cl_2 (2.5 mL) was added dropwise. The reaction mixture was stirred at 0 °C for 5 min, followed by addition of triethylamine (1.0 mL) to quench the excess CF_3COOH. After that, the resulting mixture was purified by flash column chromatography on silica gel using pentane/CH_2Cl_2 (9:1) as eluent to give the desired product ***l*-H$_2$P8**$_{Br}$ (7.2 mg, 90% yield).

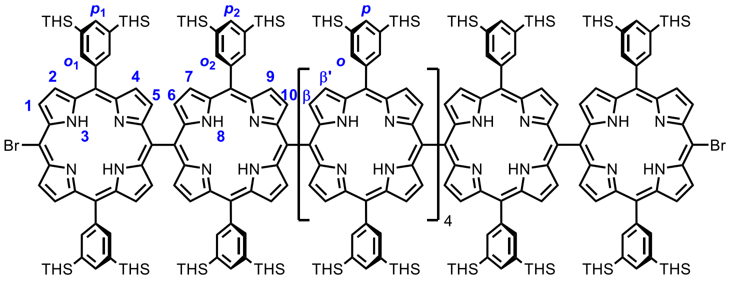

^{1}H NMR (600 MHz, $CDCl_3$, 298 K): δ 9.77 (d, J = 4.6 Hz, 4H, H_1), 8.92 (d, J = 4.6 Hz, 4H, H_2), 8.73–8.64 (m, 20H, $H_{9,\beta'}$), 8.64–8.58 (m, 8H, $H_{4,7}$), 8.37–8.32 (m, 16H, H_o), 8.32–8.26 (m, 36H, $H_{10,\beta,o1,o2}$), 8.18 (d, J = 4.7 Hz, 4H, H_5), 8.14 (d, J = 4.7 Hz, 4H, H_6), 7.94 (s, 4H, H_{p1}), 7.87–7.83 (m, 8H, H_p), 7.82 (s, 4H, H_{p2}), 1.48–0.35 (m, 1184H, H_{THS}), –1.36 – –1.65 (m, 12H, $H_{8,NH}$), –2.06 (s, 4H, H_3).

MALDI-ToF: m/z = 12889.49 ($C_{832}H_{1376}Br_2N_{32}Si_{32}$, $M^{•+}$ requires 12885.99).

UV-vis-NIR ($CDCl_3$, 298 K) λ_{max} (ε / 10^5 M^{-1} cm^{-1}): 415 (7.60), 491 (6.54), 542 (4.52), 602 (1.54), 660 (0.48) nm.

Synthesis and Characterization of *l*-NiP8$_{Br}$

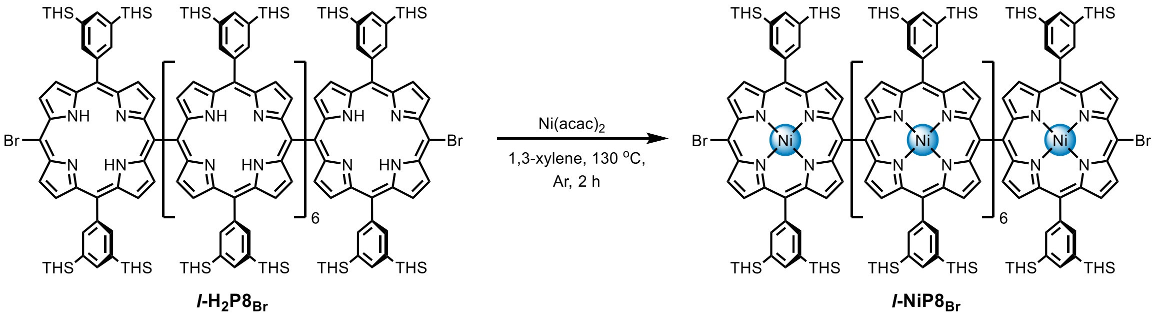

A mixture of ***l*-H$_2$P8$_{Br}$** (8.0 mg, 0.62 μmol, 1.0 equiv.) and Ni(acac)$_2$ (4.9 mg, 19 μmol, 30 equiv.) in 1,3-xylene (5.0 mL) was evacuated and backfilled with argon for three times, followed by stirring at 130 ºC under argon for 2 h. After reaction, the resulting mixture was concentrated and purified by flash column chromatography on silica gel using pentane as eluent to give the desired product ***l*-NiP8$_{Br}$** (6.5 mg, 78% yield).

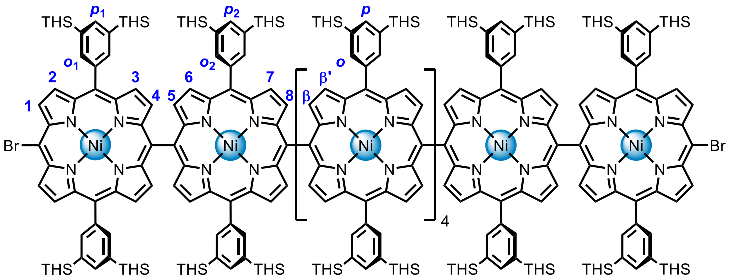

^{1}H NMR (600 MHz, CDCl$_3$, 298 K): δ 9.58 (d, J = 4.9 Hz, 4H, H_1), 8.78 (d, J = 4.9 Hz, 4H, H_2), 8.65–8.58 (m, 20H, $H_{6/7,\beta'}$), 8.59–8.53 (m, 8H, $H_{3,6/7}$), 8.28–8.20 (m, 20H, $H_{5/8,\beta}$), 8.16 (d, J = 4.9 Hz, 4H, H_4), 8.15–8.12 (m, 16H, H_o), 8.11 (d, J = 4.9 Hz, 4H, $H_{5/8}$), 8.10 – 8.09 (m, 8H, H_{o2}), 8.08–8.07 (m, 8H, H_{o1}), 7.88–7.86 (m, 4H, H_{p1}), 7.81–7.79 (m, 8H, H_p), 7.78 (s, 4H, H_{p2}), 1.45–0.49 (m, 1184H).

MALDI-ToF: m/z = 13341.61 ($C_{832}H_{1360}Br_2N_{32}Ni_8Si_{32}$, $M^{\bullet+}$ requires 13339.33).

UV-vis-NIR (CDCl$_3$, 298 K) λ_{max} (ε / 10^5 M^{-1} cm^{-1}): 413 (5.26), 482 (6.08), 551 (3.05) nm.

Synthesis and Characterization of *f*-NiP8$_{Br}$

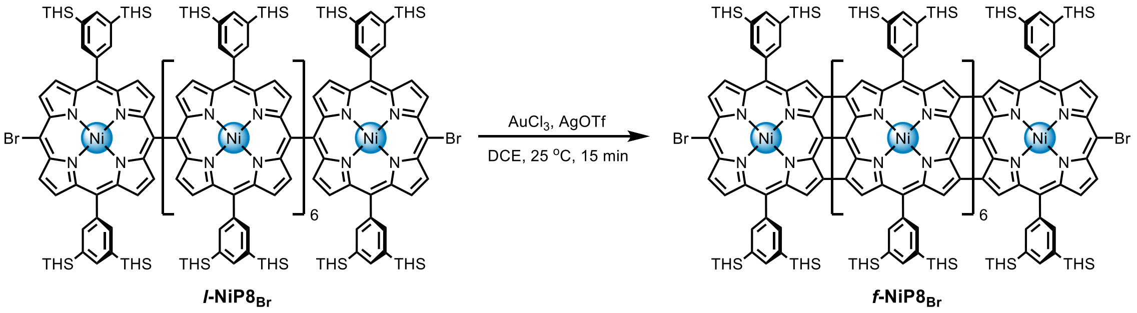

To a solution of ***l*-NiP8$_{Br}$** (2.0 mg, 0.15 μmol, 1 equiv.) in dry 1,2-dichloroethane (DCE, 2.5 mL), a suspension of $AuCl_3$ (0.64 mg, 2.1 μmol, 14 equiv.) and AgOTf (2.7 mg, 11 μmol, 70 equiv.) in dry DCE (2.5 mL) was added dropwise and the reaction mixture was stirred at 25 ºC for 15 min. After that, a suspension of $AuCl_3$ (0.13 mg, 0.42 μmol, 2.8 equiv.) and AgOTf (0.54 mg, 2.1 μmol, 14 equiv.) in dry DCE (0.5 mL) was added dropwise to the reaction mixture and the reaction was monitored by UV-Vis-NIR spectroscopy with CH_2Cl_2 + 1% triethylamine as solvent. After the completion, triethylamine (1.0 mL) was added to the reaction mixture. The resulting mixture purified by flash column chromatography on silica gel using pentane as eluent to give the desired product ***f*-NiP8$_{Br}$** (1.0 mg, 50% yield).

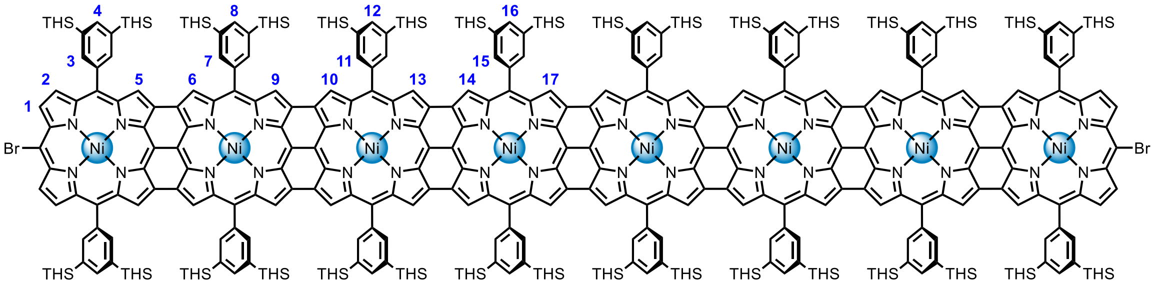

^{1}H NMR (700 MHz, CDCl$_3$, 298 K): δ 8.50 (d, *J* = 4.8 Hz, 4H, ***H$_1$***), 7.77 (s, 4H, ***H$_4$***), 7.74–7.64 (m, 20H, ***H$_{3,8,12,16}$***), 7.57 (d, *J* = 4.8 Hz, 4H, ***H$_2$***), 7.54 (s, 8H, ***H$_7$***), 7.51–7.46 (m, 16H, ***H$_{11,15}$***), 7.28 (s, 4H, ***H$_5$***), 6.72 (s, 4H, ***H$_6$***), 6.66–6.53 (m, 20H, ***H$_{9,10,13,14,17}$***), 1.40–0.70 (m, 1184H, ***H$_{THS}$***).

MALDI-ToF: *m*/*z* = 13335.34 ($C_{842}H_{1350}N_{32}Ni_8Si_{34}$, $M^{\bullet+}$ requires 13345.38).

UV-vis-NIR (CDCl$_3$, 298 K) λ_{max} (ε / 10^5 M^{-1} cm^{-1}): 412 (2.87), 776 (5.52), 1788 (7.97) nm.

Synthesis and Characterization of ***f*-NiP8**

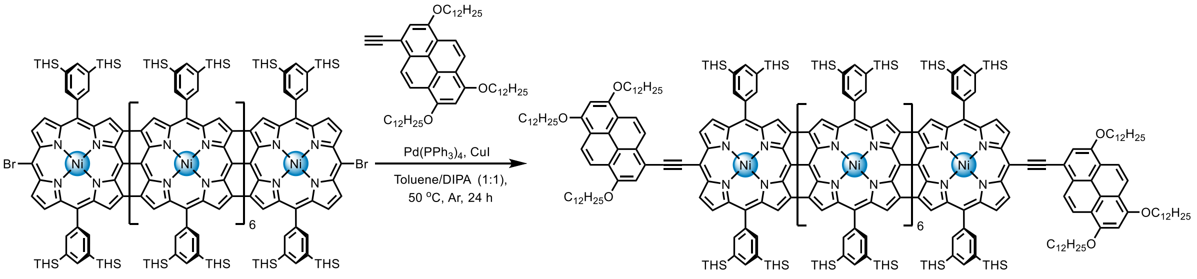

***f*-NiP8$_{Br}$** ***f*-NiP8**

A mixture of ***f*-NiP8$_{Br}$** (1.0 mg, 0.075 µmol, 1.0 equiv.), $Pd(PPh_3)_4$ (1.1 mg, 1.5 µmol, 20 equiv.) and CuI (0.14 mg, 0.75 µmol, 10 equiv.) in dry toluene (0.5 mL) and diisopropylamine (DIPA, 0.5 mL) was degassed by three freeze-pump-thaw cycles. A solution of 1,3,6-tris(dodecyloxy)-8-ethynylpyrene[2] (10 mg, 13 µmol, 170 equiv.) in dry toluene (0.5 mL) and diisopropylamine (0.5 mL) was degassed by three freeze-pump-thaw cycles and transferred to the reaction mixture under argon. After that, the mixture was stirred at 50 ºC under argon for 2 h. Then, a degassed solution of 1,3,6-tris(dodecyloxy)-8-ethynylpyrene (5.0 mg, 6.4 µmol, 85 equiv.) in dry toluene (0.5 mL) and DIPA (0.5 mL) was added to the reaction mixture and the mixture stirred at 50 ºC for another 20 h. After reaction, the resulting mixture was separated by flash column chromatography on silica gel using pentane/CH_2Cl_2 (1:1) as eluent, followed by size-exclusion chromatography (Biorad Bio beads SX-1) with toluene/pyridine (99:1) as eluent to give the crude mixture. The crude mixture was further subjected to reversible recycling GPC with toluene/pyridine (99:1) as eluent to separate the desired product ***f*-NiP8** (0.11 mg, 11%).

MALDI-TOF: *m*/*z* = 14719.70 ($C_{940}H_{1494}N_{32}Ni_8O_6Si_{32}$, $M^{\bullet+}$ requires 14707.52).

UV-vis-NIR ($CDCl_3$, 298 K): λ_{max} = 414, 770, 1728 nm.

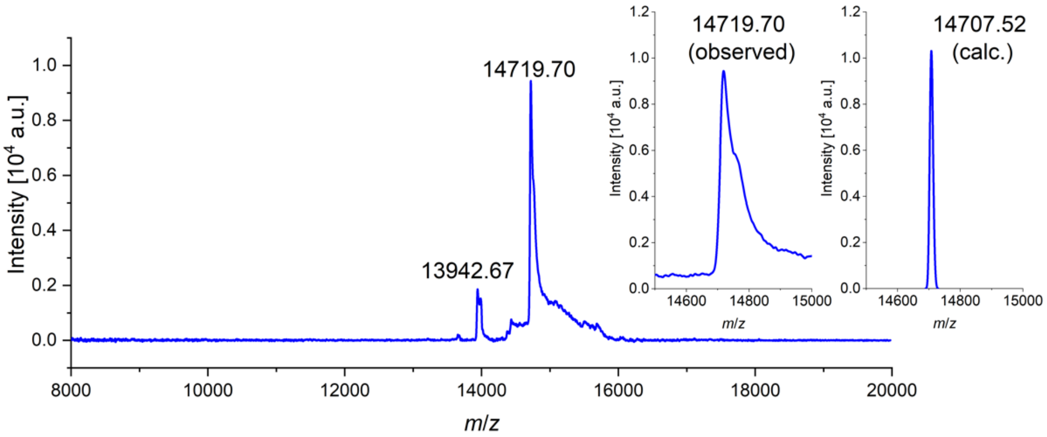

Fig. S2-1. MALDI-ToF spectrum of ***f*-NiP8** (found *m*/*z* = 13942.67, calc. $C_{940}H_{1494}N_{32}Ni_8O_6Si_{32}$: 14707.52) with DCTB as matrix. The peak found *m*/*z* = 13942.67 corresponds to singly coupled side product (calc. $C_{886}H_{1414}N_{32}Ni_8O_3Si_{32}$: 13930.91).

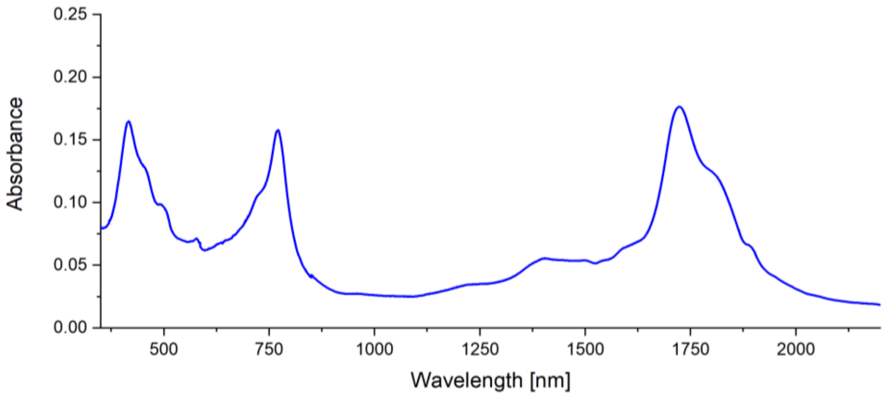

Fig. S2-2. UV-vis-NIR absorption spectrum of ***f*-NiP8** in $CDCl_3$ at 298 K.

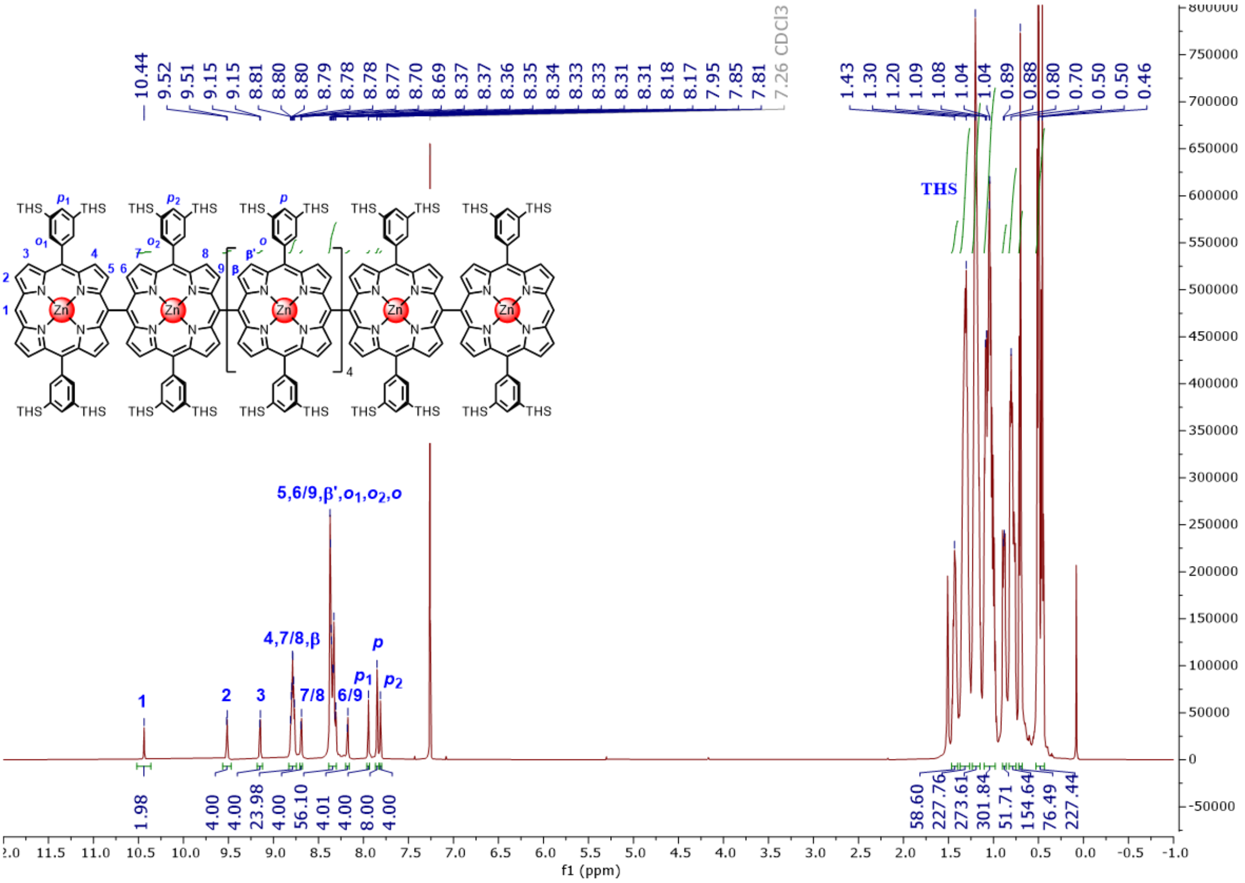

Fig. S2-3. ^{1}H NMR spectrum of ***l*-ZnP8$_H$** (600 MHz, $CDCl_3$, 298 K).

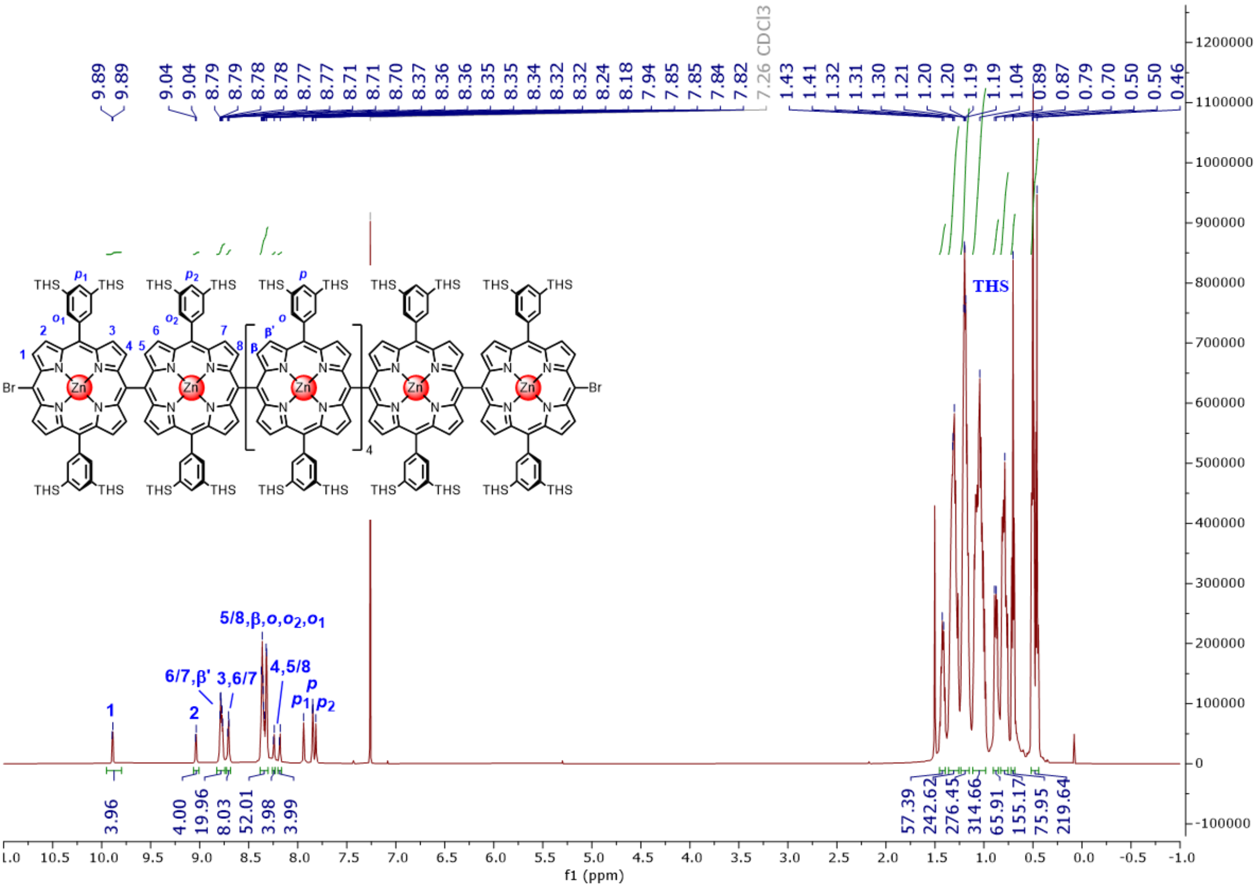

Fig. S2-4. ^{1}H NMR spectrum of ***l*-ZnP8$_{Br}$** (600 MHz, $CDCl_3$, 298 K).

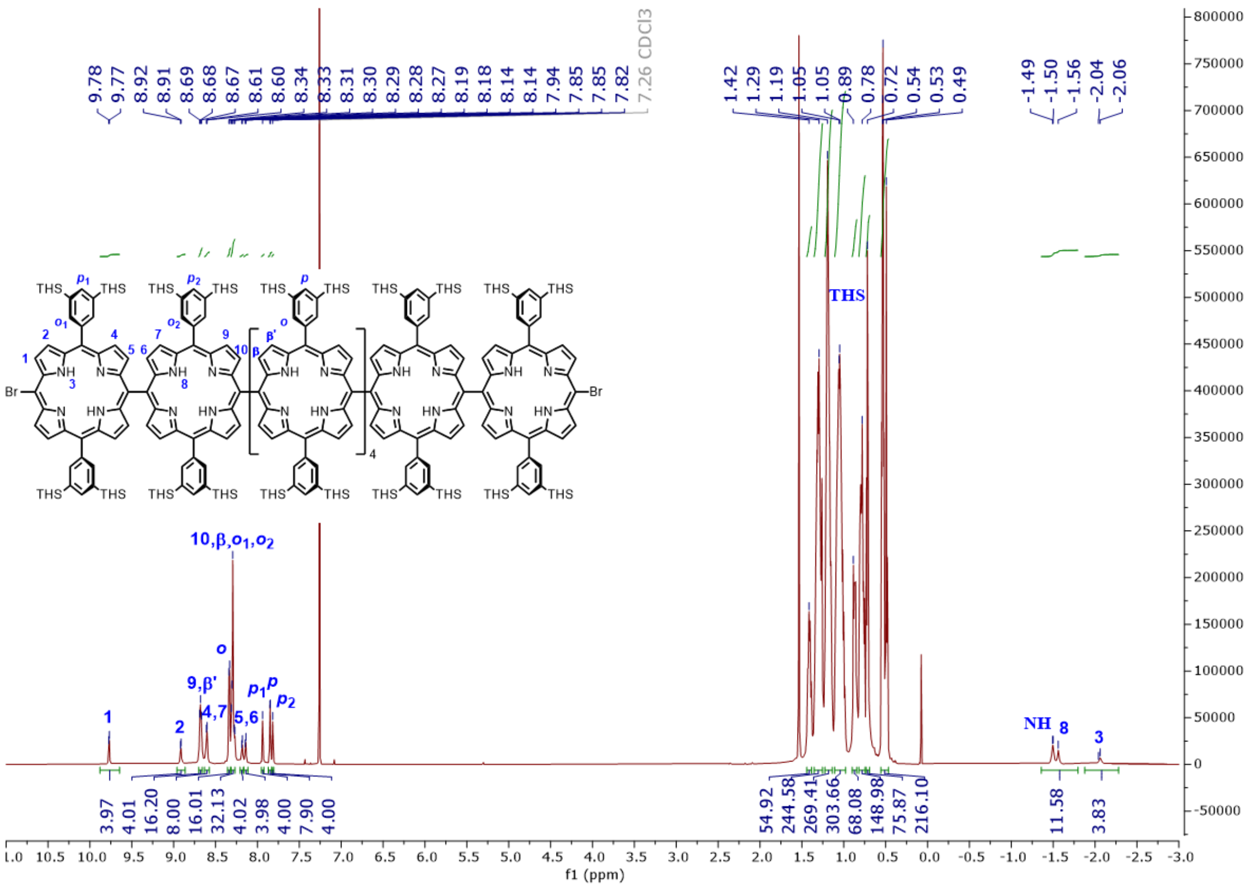

Fig. S2-5. ^{1}H NMR spectrum of ***l*-H$_2$P8$_H$** (600 MHz, $CDCl_3$, 298 K).

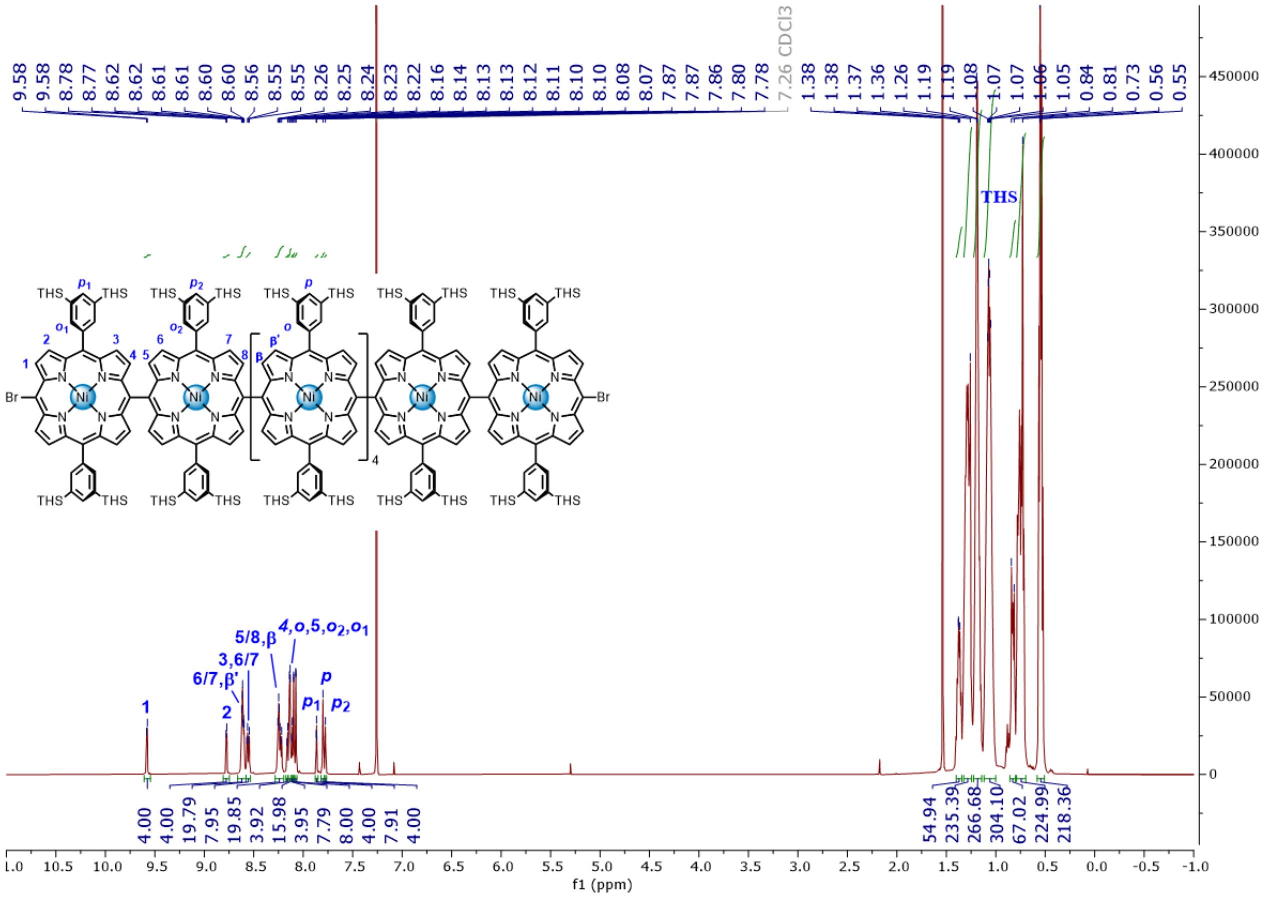

Fig. S2-6. ^{1}H NMR spectrum of ***l*-NiP8$_{Br}$** (600 MHz, $CDCl_3$, 298 K).

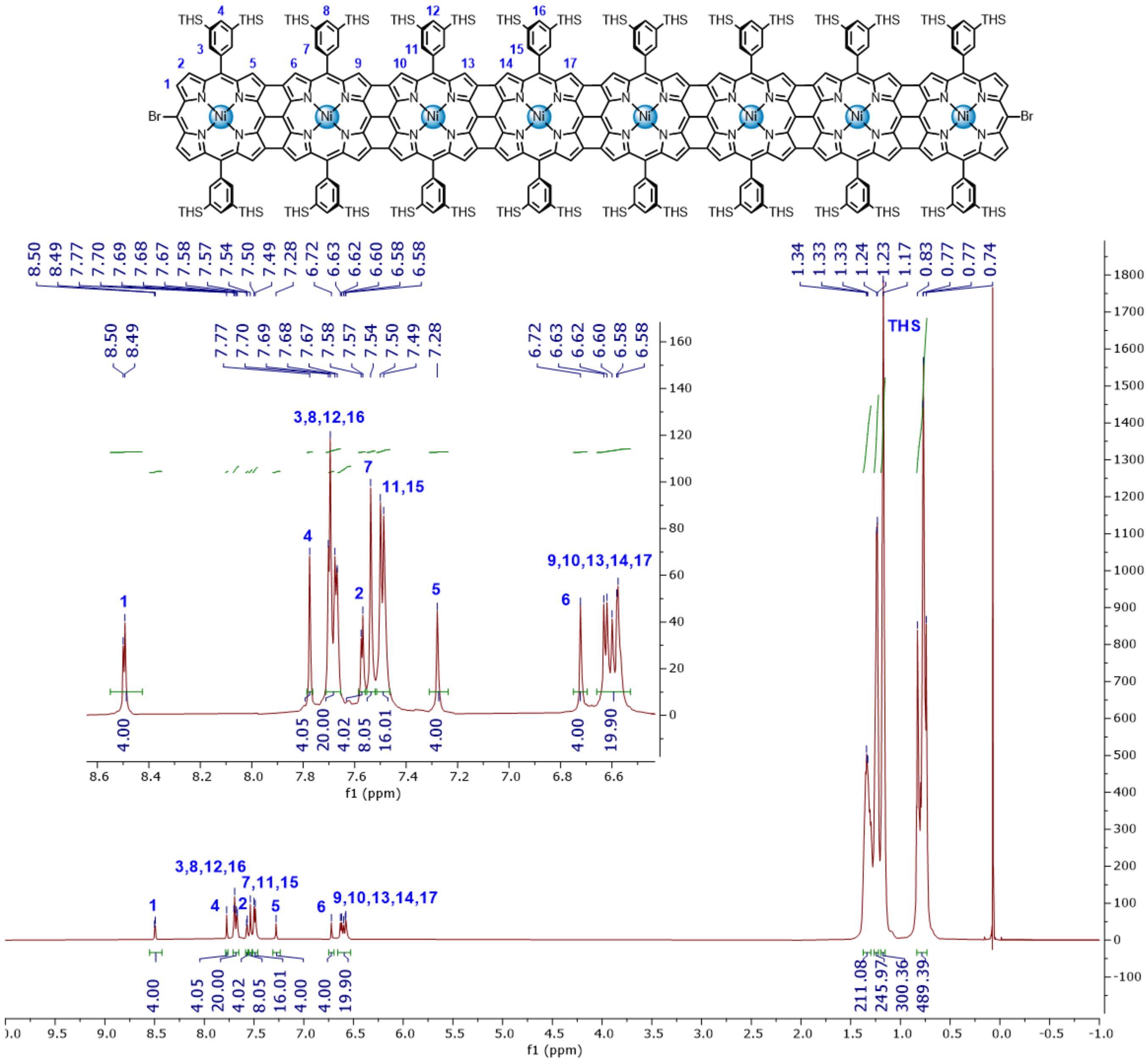

Fig. S2-7. Diffusion edited ^{1}H NMR spectrum of ***f*-NiP8$_{Br}$** (700 MHz, $CDCl_3$, 298 K).

Supplemental Section 3 Transport Measurement

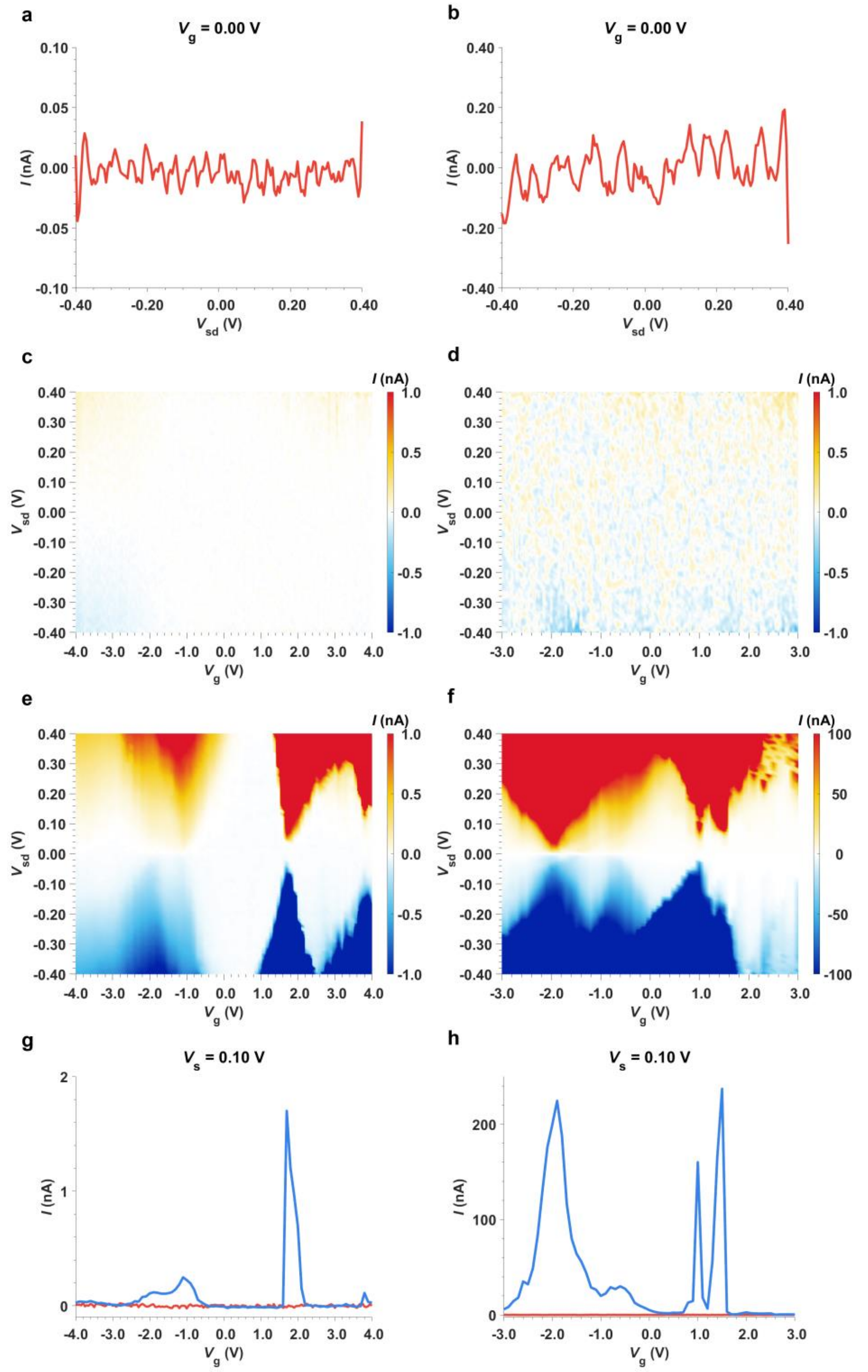

Fig. S3-1. Devices were measured before and after molecular deposition to ensure source-drain current is due to tunneling through the molecule. **a**, **b** I_{sd}-V_{sd} traces after electroburning for the two **Ni-FP8** devices (device 1 and 2). Calculation of nanogap width is usually obtained from fitting I_{sd}-V_{sd} traces to the Simmons model,[3] however due to the low current this was not possible. Based on analysis in Ref [4] we estimate a minimum gap size of 2.5 nm. **c** and **d** display current maps prior to **Ni-FP8** deposition for device 1 and device 2 respectively, and **e** and **f** display the current maps after **Ni-FP8** deposition. **g**, **h** Gate traces for the devices at fixed source-drain voltage (0.1 V) before (red) and after (blue) **Ni-FP8** deposition, displaying that the current results from transport through the molecule. Data is taken at room temperature, except for that after deposition.

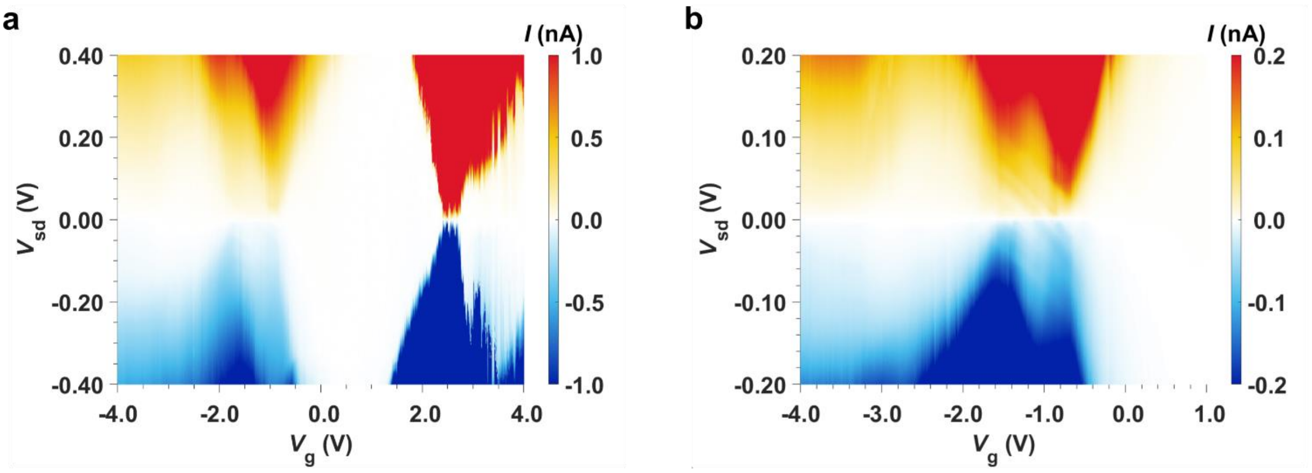

Fig. S3-2. Full-range (a) and detailed (b) current map as a function of bias voltage (V_{sd}) and gate voltage (V_g) for **Ni-FP8** device 1 measured at 4.2 K.

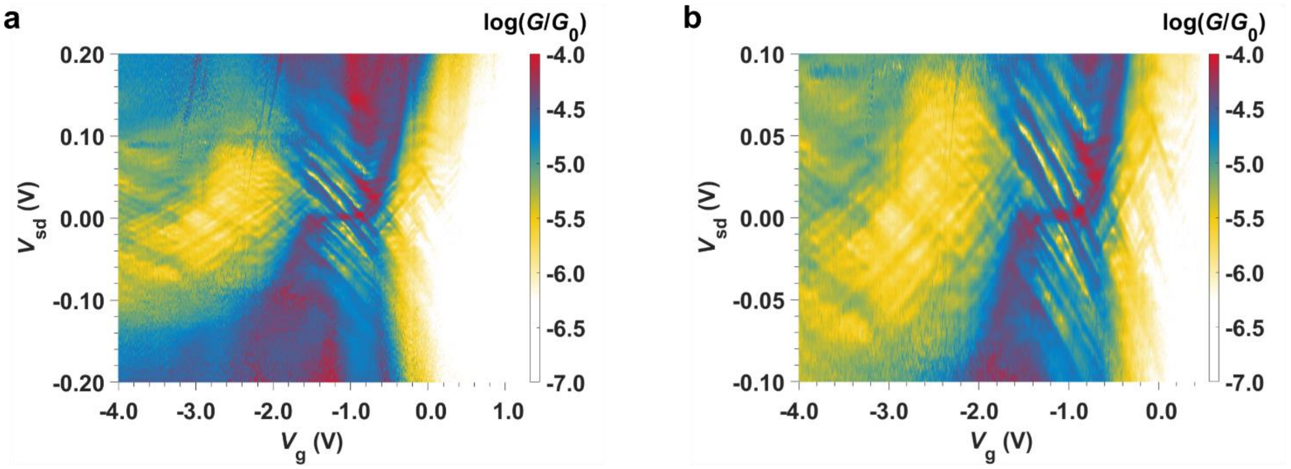

Fig. S3-3. Detailed differential conductance maps at different resolution for **Ni-FP8** device 1 measured at 4.2 K.

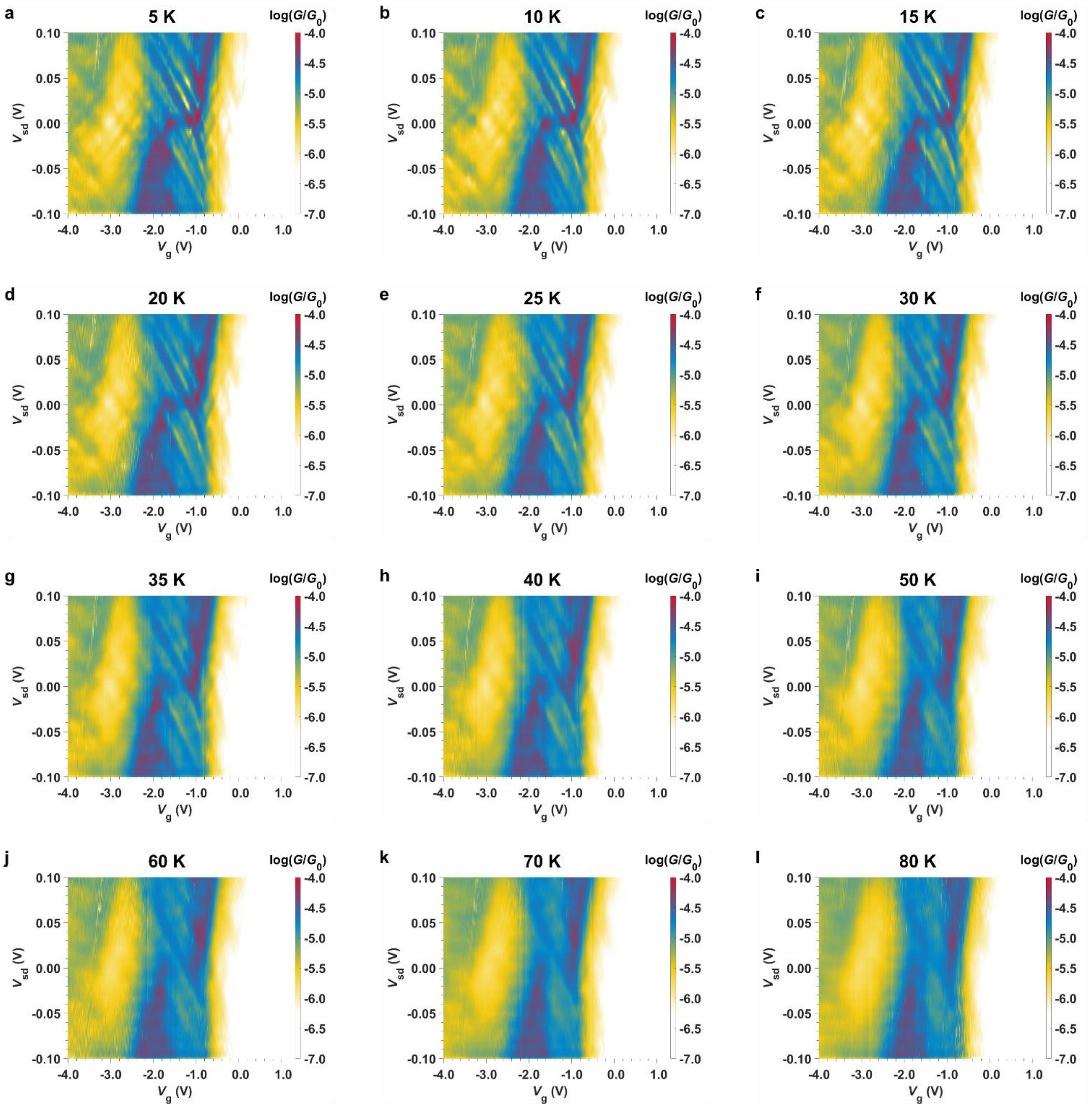

Fig. S3-4. Differential conductance map as a function of bias voltage (V_{sd}) and gate voltage (V_g) for **Ni-FP8** device 1 measured at different temperature.

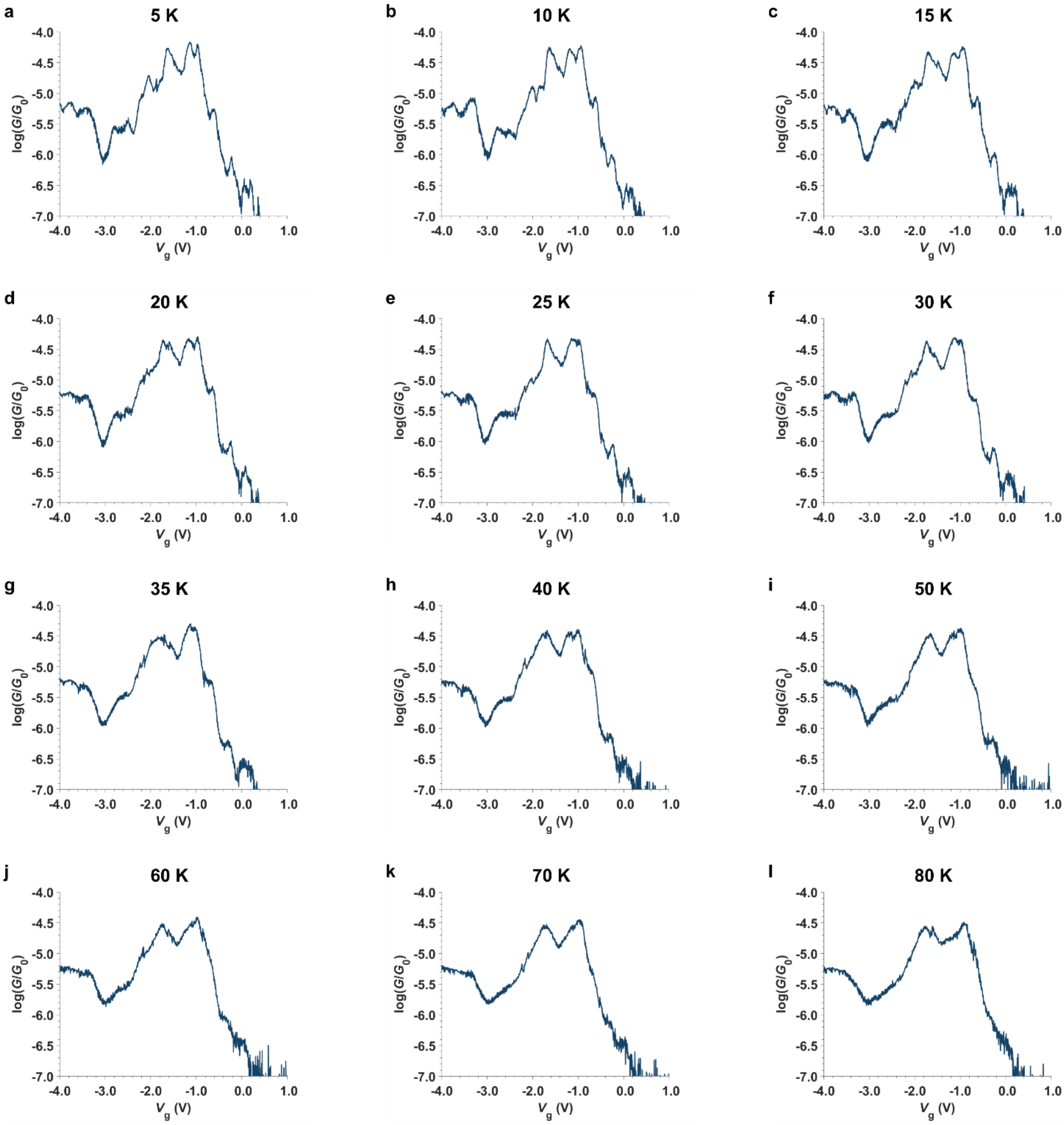

Fig. S3-5. Differential conductance as a function of gate voltage (V_g) at V_{sd} = 0 V for **Ni-FP8** device 1 measured at different temperatures.

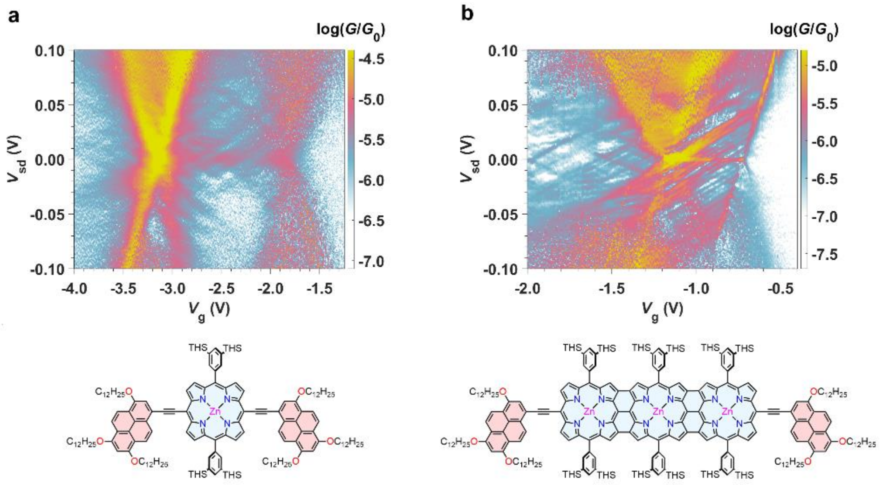

Fig. S3-6. Detailed differential conductance map of the region with interference pattern for devices with zinc porphyrin monomer (a, **Zn-P1**), with edge-fused zinc porphyrin trimer (b, **Zn-FP3**).

Supplemental Section 4 Data Analysis

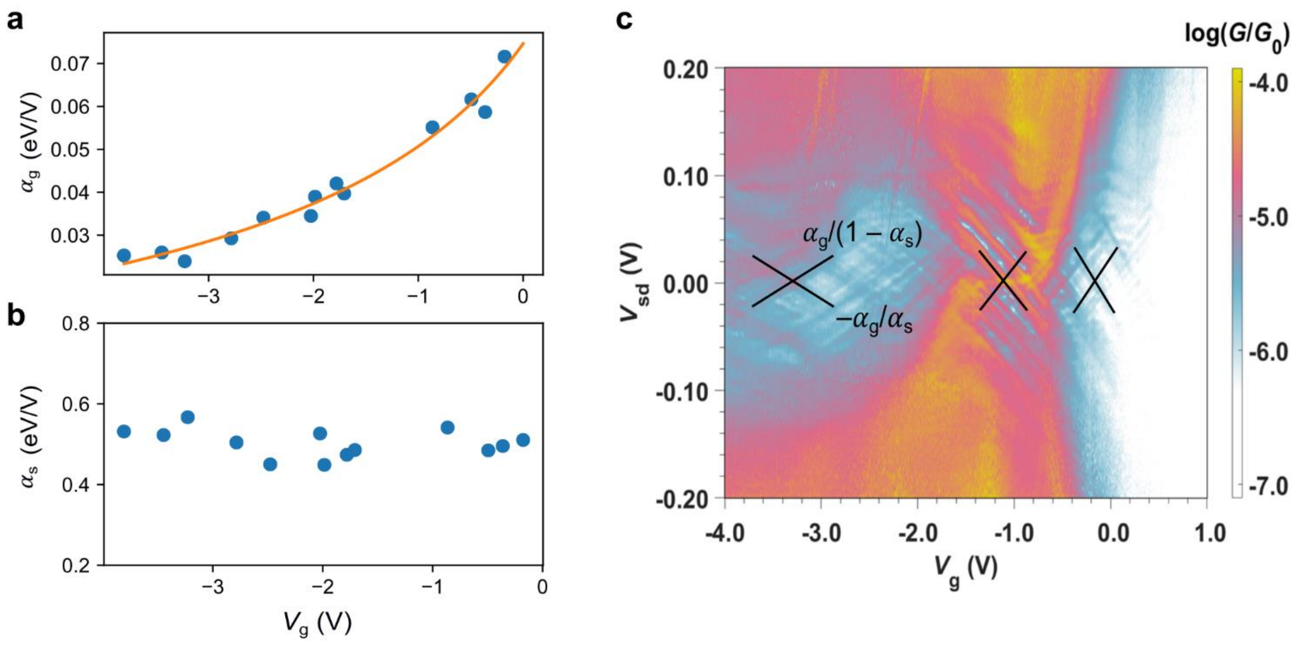

Fig. S4-1. Electrostatic coupling of FP peaks to gate (**a**) and source electrode (**b**) as a function of gate voltage. Examples of FP modes and equations[5] used to calculate the couplings are indicated on (**c**). The gate-voltage dependence of the coupling is related to the charge-carrier density of graphene, *n*, through: $\alpha_{g,FP} = \frac{dE}{dV_g} = \frac{dE}{dn}\frac{dn}{dV_g} \propto \frac{1}{\sqrt{V_g - V_{Dirac}}}$, as $E \propto \sqrt{n}$ and $n \propto (V_g - V_{Dirac})$. A fit to the data is given by the orange line in (**a**), which gives the Dirac point as V_{Dirac} = 1.6 V.

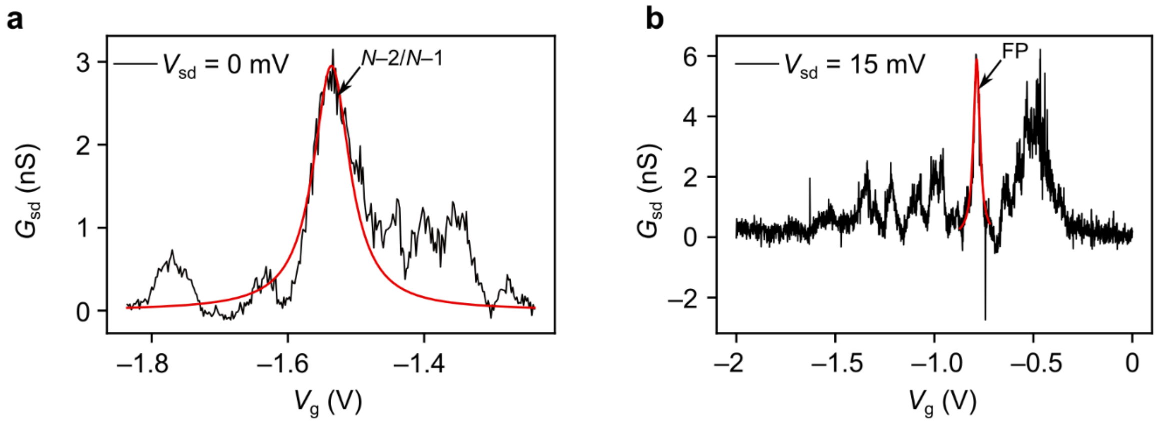

Fig. S4-2. Fitting Coulomb (**a**) and FP peaks (**b**) to Lorentzians with FWHMs of 14.1 ± 0.8 meV, and 2.1 ± 0.1 meV respectively (after scaling by their gate coupling).

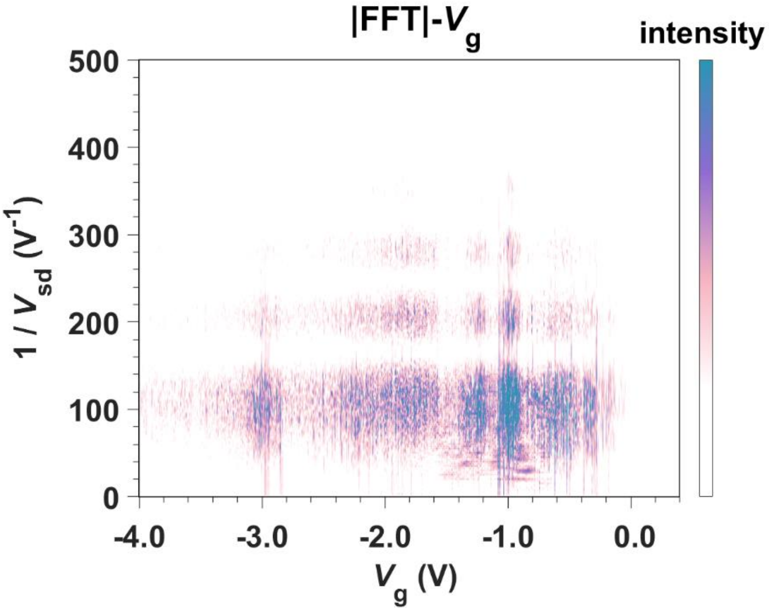

Fig. S4-3 1D fast Fourier transform (FFT) on each single $dI^2{}_{sd}/dV^2{}_{sd}$ curves of Fig. 2b, plotted as a function of V_g. The high density hot spots estimate the characteristic energy spacing to be ~ 4-5 meV and ~8–10 meV. The FFT analysis gives some higher order harmonics to as the signal is a series of Lorentzians rather than purely sinusoidal.

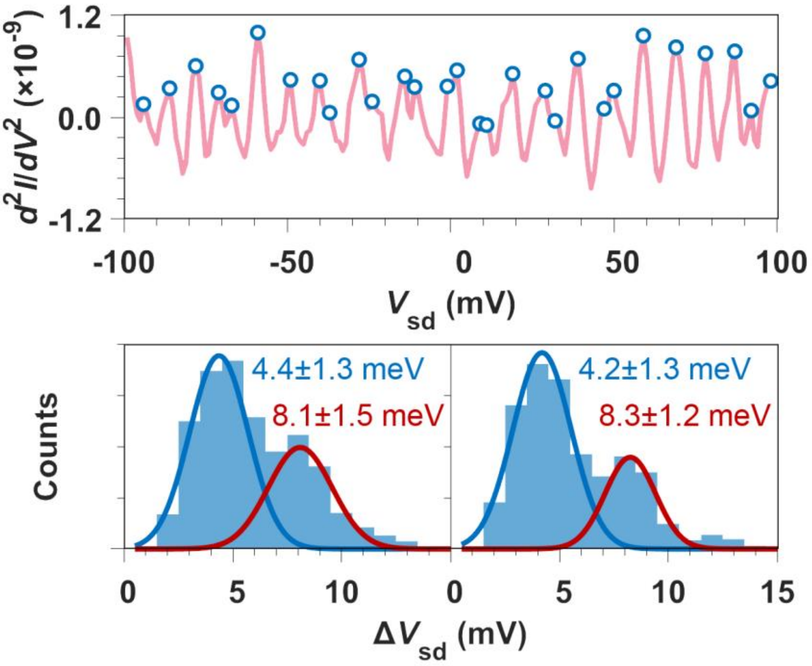

Fig. S4-4 (a) Individual $dI^2/dV_{sd}{}^2$ -V_{sd} trace at Vg = –4 V for device 1 with blue spots marking conductance peaks. (b) The energy spacings between adjacent peaks for each gate voltage are plotted in normalized histograms (total counts are 2300 and 3700) for the two **Ni-FP8** devices (device 1 - left, device 2 - right) in Figure 2.

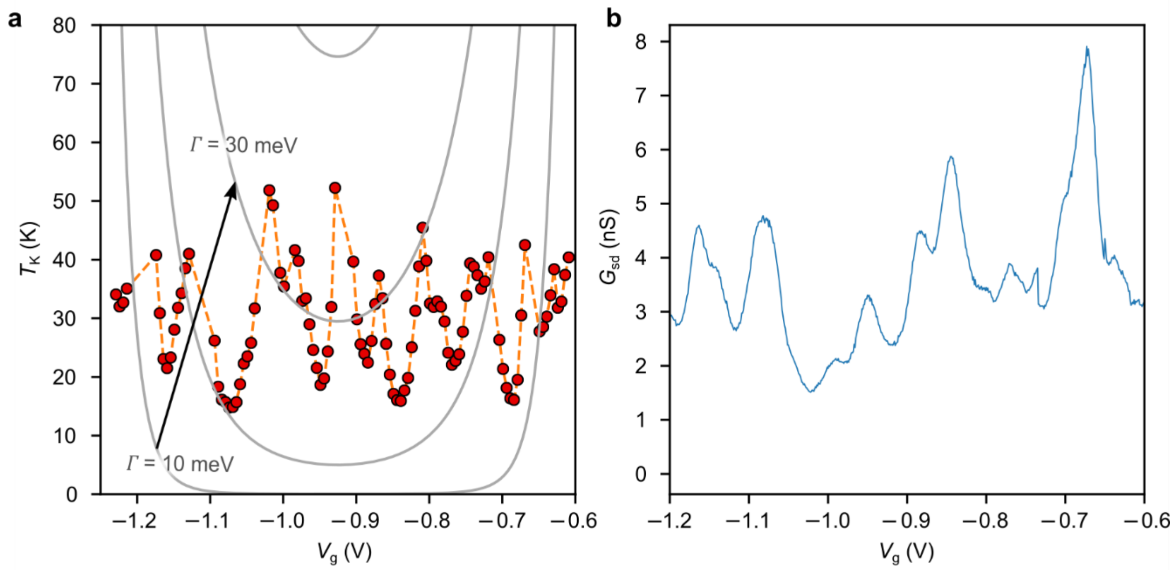

Fig. S4-5 (a) Kondo temperatures (T_K) extracted from FWHM of G-V_{sd} measurements across N–1 charge state. Grey lines indicate T_K from calculated from the Haldane relation $k_B T_K = \frac{\sqrt{\Gamma U}}{2} e^{-\pi\varepsilon_0(-\varepsilon_0+U)/\Gamma U}$ for different values of Γ, and U = 0.12 eV (the addition energy of the N–1). The gate trace is given in (b) showing the oscillation in conductance due to the coexistence of Kondo and FP channels.

Supplemental Section 5 DFT Calculation

Fig. S5-1 shows the relaxed geometry used to compute the transmission coefficient of single octamer junctions with graphene electrodes (i.e., left and right graphene leads), shown in Fig. 3 of the main text. The octamer is not quite planar, but instead assumes an undulating conformation.

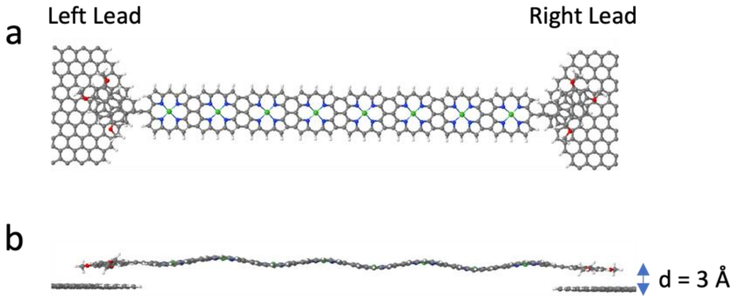

Fig. S5-1 The top view (a) and side view (b) of the graphene junction used to calculate the transmission coefficients in Fig. 3 in the main text. The distance between the anchors (two pyrene) and graphene is set to 3 Å.

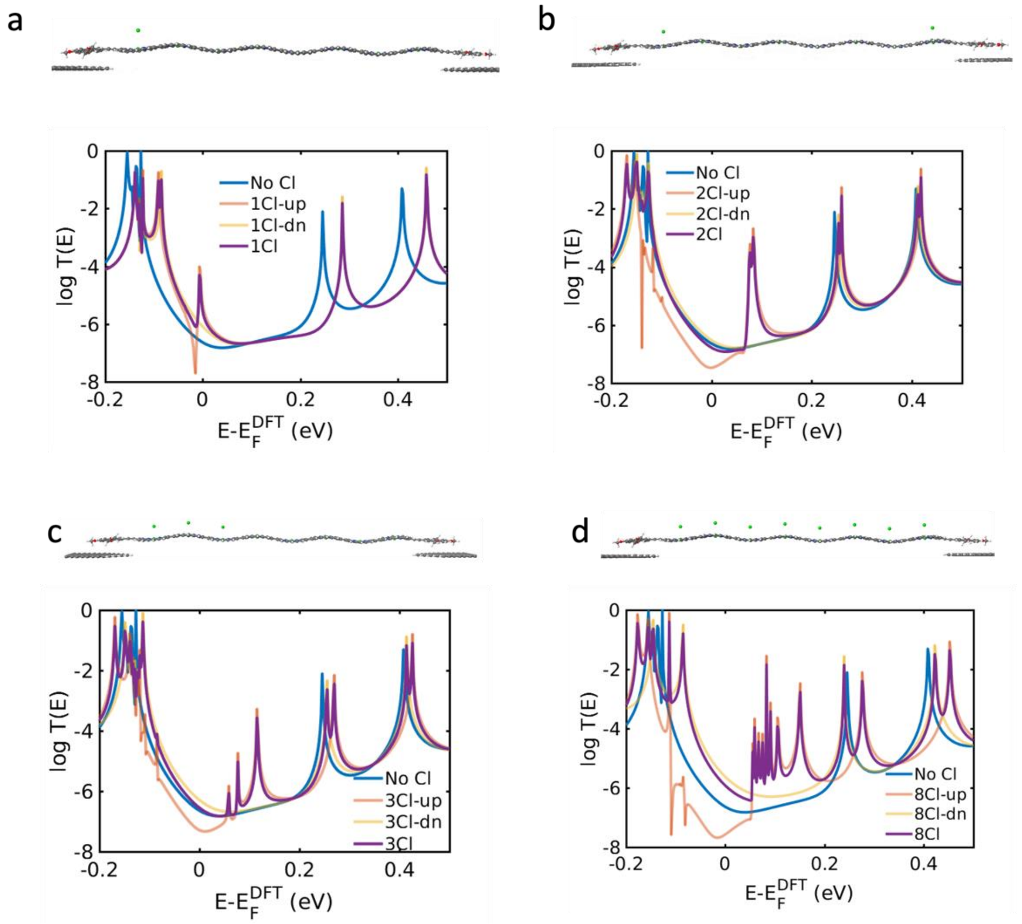

Fig. S5-2 Transmission against energy for 0, 1, 2, 3 and 8 Cl atoms. In one of the two spin-polarized transmission functions, there are one, two, three and eight extra peaks appearing when one, two, three and eight Cl atoms are present.

As well as mimicking the effect of an electrostatic gate, by oxidizing the octamer, it is interesting to note that the Cl atoms produce additional transport resonances, which are not relevant to our current experiments, (which utilize an electrostatic gate, remote from the junction), but would be relevant in an experiment in which electrochemical gating is employed. To demonstrate this feature, Fig. S5-2 shows a comparison between the transmission

coefficient of the neutral molecule (blue curve) and the transmission coefficients in the presence of 1, 2, 3 and 8 Cl atoms (purple curves in Figs. S5-2a to S5-2d respectively). Also shown are the transmission coefficients $T^{\uparrow}$, $T^{\downarrow}$ for the two different spins. All quantities are plotted against $E - E^{DFT}$, where E^{DFT} is the Fermi energy predicted by density functional theory. Fig. S5-2a shows that compared with the neutral case, in the presence of one Cl atom, a new resonance appears near $E - E^{DFT} = 0$ eV whereas in the presence of two Cl atoms, two almost-degenerate resonances appear near $E - E^{DFT} = 0.08$eV. In the presence of three Cl atoms, three new resonances occur in the range 0.05 to 0.11 eV, while Fig. S5-2d shows that the presence of eight Cls leads to eight overlapping resonances in the interval 0.05 to 0.15 eV. As shown in Fig. S5-3, these new resonances have a Fano-like characteristic and are associated with the presence of localized states on the Cl atoms.

Tab. S5-1 The Voronoi charge on Cl and octamer, positive value indicates lose electron while negative one indicates gain electron.

Charge(e^-)	1Cl	2Cl	3Cl	8Cl
Octamer	0.26	0.64	0.94	2.46
Cl	-0.33	-0.69	-0.98	-2.54

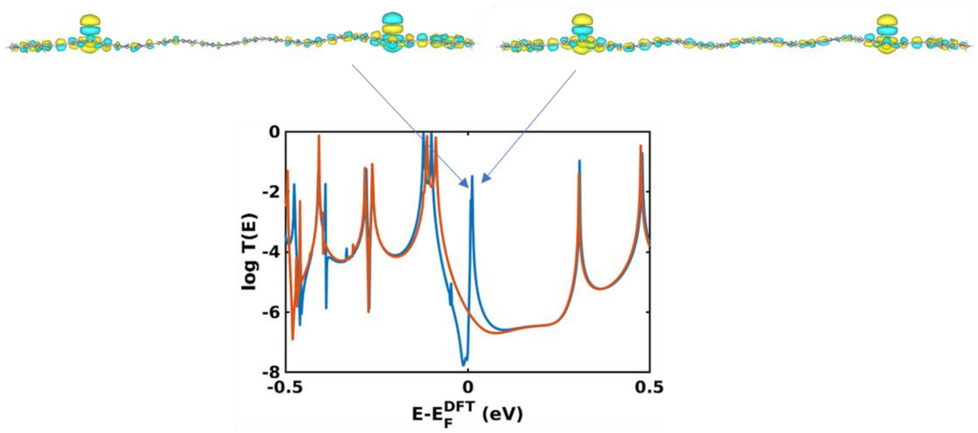

Fig. S5-3 In the case of 2 Cl atoms, the two new resonances are associated with the two Cl atoms. The corresponding two frontier orbitals of gas phase octamer with two Cl atoms are indicated by the two blue arrow.

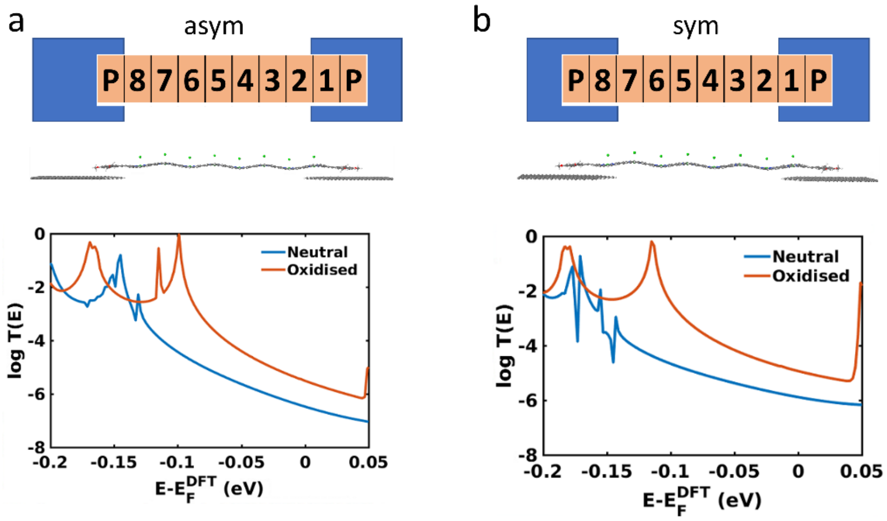

Fig. S5-4 Transmission against energy for neutral and oxidized **Ni-FP8** with different anchoring symmetry. (a) Schematic and real model used to obtain the transmission functions shown in bottom panel for asymmetric case. (b) Schematic and real model used to obtain the transmission functions shown in bottom panel for symmetric case.

In the schematic junction, 'p' denotes the pyrene anchors while the eight porphyrins are presented by numbers from 1 to 8. It should be noted that the 'symmetric' junction, in this context, is not exactly symmetric due to the slight changes in the atomic contact details between molecule and the two graphene electrodes.

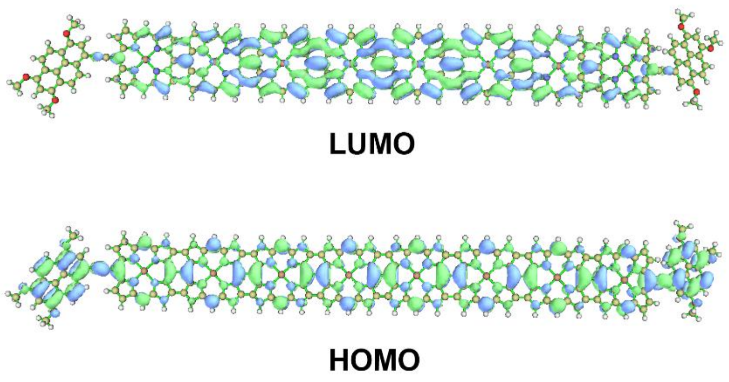

Fig. S5-5 DFT-calculated frontier molecular orbitals (isovalue = 0.01) of **Ni-FP8**. *Bis*(3,5-trihexylsilyl)phenyl groups, and $C_{12}H_{25}$ chains have been replaced by H atoms and methyl groups on the porphyrins and pyrene anchoring groups respectively. The geometry was optimized in Gaussian, with a B3LYP/6-31G(d) functional/basis set combination.[6]

Supplemental References

1. Richert, S.; Limburg, B.; Anderson, H. L.; Timmel, C. R., On the Influence of the Bridge on Triplet State Delocalization in Linear Porphyrin Oligomers. *J. Am. Chem. Soc.* **2017,** *139* (34), 12003-12008.
2. Limburg, B.; Thomas, J. O.; Holloway, G.; Sadeghi, H.; Sangtarash, S.; Hou, I. C. Y.; Cremers, J.; Narita, A.; Müllen, K.; Lambert, C. J.; Briggs, G. A. D.; Mol, J. A.; Anderson, H. L., Anchor groups for graphene-porphyrin single-molecule transistors. *Adv. Funct. Mater.* **2018,** *28* (45), 1803629.
3. Prins, F.; Barreiro, A.; Ruitenberg, J. W.; Seldenthuis, J. S.; Aliaga-Alcalde, N.; Vandersypen, L. M. K.; van der Zant, H. S. J., Room-Temperature Gating of Molecular Junctions Using Few-Layer Graphene Nanogap Electrodes. *Nano Lett.* **2011,** *11* (11), 4607-4611.
4. Limburg, B.; Thomas, J. O.; Holloway, G.; Sadeghi, H.; Sangtarash, S.; Hou, I. C.-Y.; Cremers, J.; Narita, A.; Müllen, K.; Lambert, C. J.; Briggs, G. A. D.; Mol, J. A.; Anderson, H. L., Anchor Groups for Graphene-Porphyrin Single-Molecule Transistors. *Adv. Funct. Mater.* **2018,** *28* (45), 1803629.
5. Osorio, E.; Bjørnholm, T.; Lehn, J.; Ruben, M.; Van der Zant, H., Single-molecule transport in three-terminal devices. *J. Phys.: Condens. Matter* **2008,** *20* (37), 374121.
6. Frisch, M. J.; Trucks, G. W.; Schlegel, H. B.; Scuseria, G. E.; Robb, M. A.; Cheeseman, J. R.; Scalmani, G.; Barone, V.; Petersson, G. A.; Nakatsuji, H.; Li, X.; Caricato, M.; Marenich, A. V.; Bloino, J.; Janesko, B. G.; Gomperts, R.; Mennucci, B.; Hratchian, H. P.; Ortiz, J. V.; Izmaylov, A. F.; Sonnenberg, J. L.; Williams; Ding, F.; Lipparini, F.; Egidi, F.; Goings, J.; Peng, B.; Petrone, A.; Henderson, T.; Ranasinghe, D.; Zakrzewski, V. G.; Gao, J.; Rega, N.; Zheng, G.; Liang, W.; Hada, M.; Ehara, M.; Toyota, K.; Fukuda, R.; Hasegawa, J.; Ishida, M.; Nakajima, T.; Honda, Y.; Kitao, O.; Nakai, H.; Vreven, T.; Throssell, K.; Montgomery Jr., J. A.; Peralta, J. E.; Ogliaro, F.; Bearpark, M. J.; Heyd, J. J.; Brothers, E. N.; Kudin, K. N.; Staroverov, V. N.; Keith, T. A.; Kobayashi, R.; Normand, J.; Raghavachari, K.; Rendell, A. P.; Burant, J. C.; Iyengar, S. S.; Tomasi, J.; Cossi, M.; Millam, J. M.; Klene, M.; Adamo, C.; Cammi, R.; Ochterski, J. W.; Martin, R. L.; Morokuma, K.; Farkas, O.; Foresman, J. B.; Fox, D. J. *Gaussian 16 Rev. C.01*, Wallingford, CT, 2016.
